# Continuous Versus Short EEG After Ischemic Stroke: What cEEG Adds for Detecting Abnormalities and Predicting Post‐Stroke Epilepsy

**DOI:** 10.1002/ana.78251

**Published:** 2026-05-17

**Authors:** Kai Michael Schubert, Vijaya Dasari, Chiara Tatillo, Gilles Naeije, Sándor Beniczky, Carla Bentes, Marian Galovic, Nicolas Gaspard, Vineet Punia

**Affiliations:** ^1^ Department of Neurology, Clinical Neuroscience Center University Hospital and University of Zurich Zurich Switzerland; ^2^ Epilepsy Center, Cleveland Clinic Cleveland OH; ^3^ Department of Neurology Hôpital Universitaire de Bruxelles – Hôpital Erasme Brussels Belgium; ^4^ Department of Clinical Neurophysiology Aarhus University Hospital, Aarhus and Danish Epilepsy Centre Dianalund Denmark; ^5^ Department of Neurosciences and Mental Health (Neurology), Hospital de Santa Maria – ULSSM, Centro de Estudos Egas Moniz, Faculdade de Medicina Universidade de Lisboa Lisbon Portugal; ^6^ Laboratory of Experimental Neurology Université Libre de Bruxelles Brussels Belgium; ^7^ Department of Neurology Yale University School of Medicine New Haven CT

## Abstract

**Objective:**

The objective of this study was to quantify incremental diagnostic yield and prognostic value of continuous electroencephalography (cEEG; ≥12 hours) versus a 60‐minute short electroencephalography (sEEG) in predicting post‐stroke epilepsy (PSE) in patients without acute symptomatic seizures.

**Methods:**

We retrospectively included 283 adults who underwent cEEG within 7 days; sEEG comprised the first 60 minutes of the same recording. EEGs were interpreted using American Clinical Neurophysiology Society (ACNS) terminology by neurophysiologists blinded to outcomes. Within‐patient yield was quantified using odds ratios (ORs) with 95% confidence intervals (CIs). PSE were modeled using Fine–Gray competing‐risks regression (death as competing event) and reported as subdistribution hazard ratios (sHR). SeLECT‐EEG derived from sEEG and cEEG was compared using C‐index and net reclassification improvement (NRI).

**Results:**

Over a median follow‐up of 41 months (interquartile range [IQR] = 22–64), 41 of 283 patients (14.5%) developed PSE. Compared to sEEG, cEEG increased detection of interictal epileptiform discharges (11 vs 3%, OR = 3.75, 95% CI = 1.75–8.02, *p* < 0.001) and electrographic seizures (4 vs 0.7%, OR = 6.22, 95% CI = 1.38–28.06, *p* = 0.01). Lateralized periodic discharges (sHR = 4.50, 95% CI = 2.13–9.51) and electrographic seizures (sHR = 3.63, 95% CI = 1.52–8.63) were the strongest predictors of PSE. The cEEG‐derived SeLECT‐EEG improved discrimination versus sEEG‐derived scoring (ΔC‐index 0.055, 95% CI = 0.012–0.101, *p* = 0.014) and reclassification (NRI = 0.25, 95% CI = 0.07–0.42). Epileptiform activity emerging after the first hour conferred higher 5‐year PSE risk than never detected (28 vs 11%, Gray *p* = 0.006).

**Interpretation:**

The cEEG identifies additional epileptiform abnormalities with prognostic value beyond routine‐duration EEG, supporting extension of monitoring in selected cases based on baseline risk and early EEG findings. ANN NEUROL 2026;100:400–415

Stroke is the leading cause of epilepsy in older adults and accounts for over half of new‐onset epilepsy cases in individuals aged 65 years and older.^1^ Early identification of stroke survivors at high risk of post‐stroke epilepsy (PSE) is critical to guiding follow‐up care, counseling, and future trials of preventive therapies.^2^, ^3^ Whereas clinical acute symptomatic seizures (ASyS) are a known and important risk factor,^4^, ^5^, ^6^ they occur in only a minority of patients who later develop epilepsy, highlighting the need for additional prognostic tools.^7^

Electroencephalography (EEG) is a widely available and noninvasive tool that captures early cortical dysfunction and epileptic activity.^8^ In our recently published SeLECT‐EEG study,^7^ we demonstrated that EEG findings within 7 days post‐stroke, specifically epileptiform activity and regional slowing, are strong, independent predictors of PSE, particularly in patients without ASyS. Incorporating these findings into an updated clinical model (SeLECT‐EEG) significantly improved risk stratification compared with non‐EEG‐based models in those without ASyS.^7^

However, the optimal EEG duration for prognostication remains uncertain. Although short EEGs (sEEGs = 20–60 minutes) are commonly used and logistically feasible,^9^ continuous EEGs (cEEGs; ≥12 hours) offer higher sensitivity for detecting transient abnormalities.^10^, ^11^ The added value of a cEEG over a sEEG in the context of long‐term seizure risk prediction has not been systematically investigated in post‐stroke survivors.

## Methods

### Study Cohort

We retrospectively included 283 adults with neuroimaging‐confirmed acute ischemic stroke from 2 SeLECT‐EEG centers (Hôpital Universitaire de Bruxelles, Brussels, Belgium, and Cleveland Clinic, Cleveland, OH, USA) who underwent cEEG within 7 days of stroke onset. We excluded patients with prior seizures/epilepsy, transient ischemic attack, primary hemorrhagic stroke, major epileptogenic comorbidities, and any ASyS per International League Against Epilepsy (ILAE) criteria.^12^ Center‐specific cohort definitions and cEEG indications, as well as site‐level denominators for cEEG eligibility are provided in the Supplementary Methods.

To contextualize prioritization implications at scale and to derive registry‐level estimates in our prespecified “full‐cohort” analyses, we additionally leveraged the full SeLECT consortium registry (n = 4,552, of 9 international subcohorts), including the ASyS‐excluded subset (n = 4,319), as described in the Supplement and prior SeLECT publications (SeLECT‐EEG).^7^

### Short versus Continuous EEG

In the Brussels cohort (2015–2019), cEEG was obtained for non‐lacunar supratentorial strokes with National Institutes of Health Stroke Scale (NIHSS) >8 or unexplained early neurological deterioration. In the US cohort (2012–2018), cEEG was typically initiated for unexplained altered mental status or motor events that were not clearly convulsive ASyS. To ensure a strictly controlled within‐recording comparison, the sEEG used in this study was simulated as the first 60 minutes of the very same cEEG recording (identical montage, environment, and clinical context). We used a 60‐minute cutoff for “sEEG” because the only randomized controlled trial comparing routine EEG with cEEG included two 20‐minute (total 40 minutes) routine EEGs.^13^ Additionally, the 2HELPS2B score was validated specifically on the first 60 minutes, which experts recommend as the minimum duration in resource‐poor in‐patient settings.^14^ Thus, a 60‐minute recording represents the evidence‐supported and clinically justified benchmark for comparison with cEEG. The “sEEG” and “cEEG” in our study differed only by duration of sampling. The electrode setup, clinical state, and acquisition conditions were otherwise identical.

### EEG Findings

All EEGs were interpreted by board‐certified clinical neurophysiologists using American Clinical Neurophysiology Society (ACNS) terminology.^15^ Readers were blinded to clinical outcomes and, for paired analyses, blinded to the other modality's findings (ie, sEEG vs cEEG annotations were adjudicated independently). Abnormalities were recorded as: generalized slowing; regional slowing; generalized rhythmic delta activity (GRDA); lateralized rhythmic delta activity (LRDA); sporadic interictal epileptiform discharges (IEDs); lateralized periodic discharges (LPDs); generalized periodic discharges (GPDs); electrographic seizures; and electrographic status epilepticus (SE). Baseline clinical and EEG characteristics were summarized as counts/percentages or medians with interquartile ranges (IQR).

### Prognostic Model

SeLECT is a well‐established clinical score for estimating the risk of PSE after ischemic stroke based on stroke severity, large‐artery atherosclerosis (etiology), early/acute symptomatic seizures (excluded here), cortical involvement, and territory (middle cerebral artery). SeLECT_2.0_ is an updated calibration we use for clinical prescreening. SeLECT‐EEG extends this framework by adding 2 EEG biomarkers, epileptiform activity (EA) and regional slowing. It independently improves PSE prediction, particularly in patients without ASyS. Accordingly, these are the 2 EEG findings we additionally target for detection in this study. EAs included IEDs, LRDA, LPDs, GPDs, electrographic seizures, and electrographic SE (ACNS/Salzburg criteria).^15^, ^16^, ^17^

### Statistical Analysis

The study workflow and 4 prespecified statistical analyses are summarized in Supplementary Figure S1.

First, we compared cEEG and sEEG findings within patients. For each ACNS category and SeLECT‐EEG targets (regional slowing and EA), we constructed 2 × 2 tables (present/absent on sEEG vs cEEG). We reported modality‐specific prevalences with Wilson 95% confidence intervals (CIs). Odds ratios (ORs) for cEEG versus sEEG were estimated using 2‐sided Fisher's exact tests, applying the Haldane–Anscombe 0.5 correction for zero values. The CIs were calculated on the log‐OR scale. We also summarized SeLECT‐EEG targets into 4 paired states: “neither,” “sEEG‐only,” “cEEG‐only,” and “both.”

Second, to link detected patterns to long‐term risk, we analyzed PSE in a competing‐risks framework, with death as the competing event. Cumulative incidence functions (CIFs) were estimated, and strata were compared using Gray's test. We also explored SeLECT‐EEG targets and timing effects (EEG on day 0 vs > day 0).^5^ For adjusted inference, we used Fine–Gray subdistribution hazard models, including SeLECT_2.0_ variables (NIHSS, cortical involvement, middle cerebral artery [MCA] territory infarction, and large‐artery atherosclerosis), age, and sex. Results were reported as subdistribution hazard ratios (sHRs) with 95% CIs. We compared the prognostic performance of SeLECT‐EEG computed from sEEG versus cEEG using Harrell's C‐index with bootstrap CIs, the between‐model difference (ΔC), and category‐based net reclassification improvement (NRI) across SeLECT‐EEG tertiles (low/intermediate/high; 1,000 bootstrap replicates).

Third, to develop a clinical prioritization tool for EEG monitoring in post‐stroke patients, we focused on identifying individuals who would benefit from transitioning from sEEG to cEEG. We estimated the probability of detecting new abnormalities on cEEG after a negative sEEG – as patients with positive sEEG had already shown these findings – for electrographic seizures/SE and for the broader range of EA. We developed 2 models: the first model focused strictly on detecting electrographic seizures/SE, whereas the second model was more inclusive, covering more forms of epileptiform activity (IED/LPDs/electrographic seizures/SE model: including also IEDs and LPDs). For both models, we used sEEG findings, such as regional and generalized slowing, LRDA, LPDs, and IEDs as potential predictors, provided these findings were not part of the outcome. The coefficients from these models were converted into a points‐based score and calibrated using a one‐parameter logistic fit. Model performance was evaluated using receiver operating characteristic (ROC) curves, area under the curve (AUC), decision‐curve analysis, 10 × 5‐fold cross‐validation medians, calibration (binned observed vs predicted with Wilson CIs and locally estimated scatterplot smoothing [LOESS] overlay), and precision‐recall with average precision.

Fourth, to create a prognostic prioritization rule, we aimed to determine when extending from sEEG to cEEG would improve predictions for later PSE. We focused on two key EEG findings – regional slowing EA – which are known SeLECT‐EEG variables that serve as predictors. First, we quantified the expected benefit (Egain) of extending from sEEG to cEEG. This was calculated by estimating 2 probabilities: the likelihood of detecting new EA and/or regional slowing on cEEG when it was absent on the sEEG. These probabilities were further stratified by SeLECT_2.0_ score (<4 vs ≥4); the ≥4 threshold was selected as a prespecified “high‐risk” cutoff point because it maximized the Youden index (balancing sensitivity and specificity for future PSE) in the full SeLECT registry (Supplementary Table S1). Second, using the results from the first step, we computed the risk of developing PSE for each sEEG pattern and SeLECT subgroup. This led to the creation of an PSE Egain matrix, where one axis represented the expected benefit of extending to cEEG (Egain) and the other represented the risk of PSE. This matrix helped identify which EEG findings would provide the highest diagnostic yield when extending the recording duration, while also being clinically relevant for predicting PSE. The matrix divided patients into 4 categories: high gain with high risk, high gain with lower risk, low gain with high risk, and low gain with lower risk.

For all cases, PSE, follow‐up time, death, and all covariates used in adjusted models were complete (no missing data). Analyses were run in R software (version 4.x) using cmprsk and riskRegression for competing risks (Fine–Gray, predictRisk); survival/Hmisc for C‐index; pROC for AUROC (DeLong), and PRROC for precision–recall curve with Average Precision; boot for resampling; binom for Wilson 95% CIs; data handling via dplyr, tidyr, purrr, forcats, and readxl; tables via writexl/openxlsx and coefficient tidying via broom; graphics with ggplot2 plus cowplot, ggrepel, scales, and colorspace.

## Results

Among 283 stroke survivors without clinical ASyS, 41 (14.5%) developed PSE over a median 41 months (IQR = 22–64 months). The median age was 64 years (IQR = 55–74 years), and 51% were men. Baseline characteristics, stroke features, and outcome comparisons (including hazard estimates) are summarized in Table 1 and Supplementary Table S2.

### Detection Yield of cEEG versus sEEG

**FIGURE 1:**
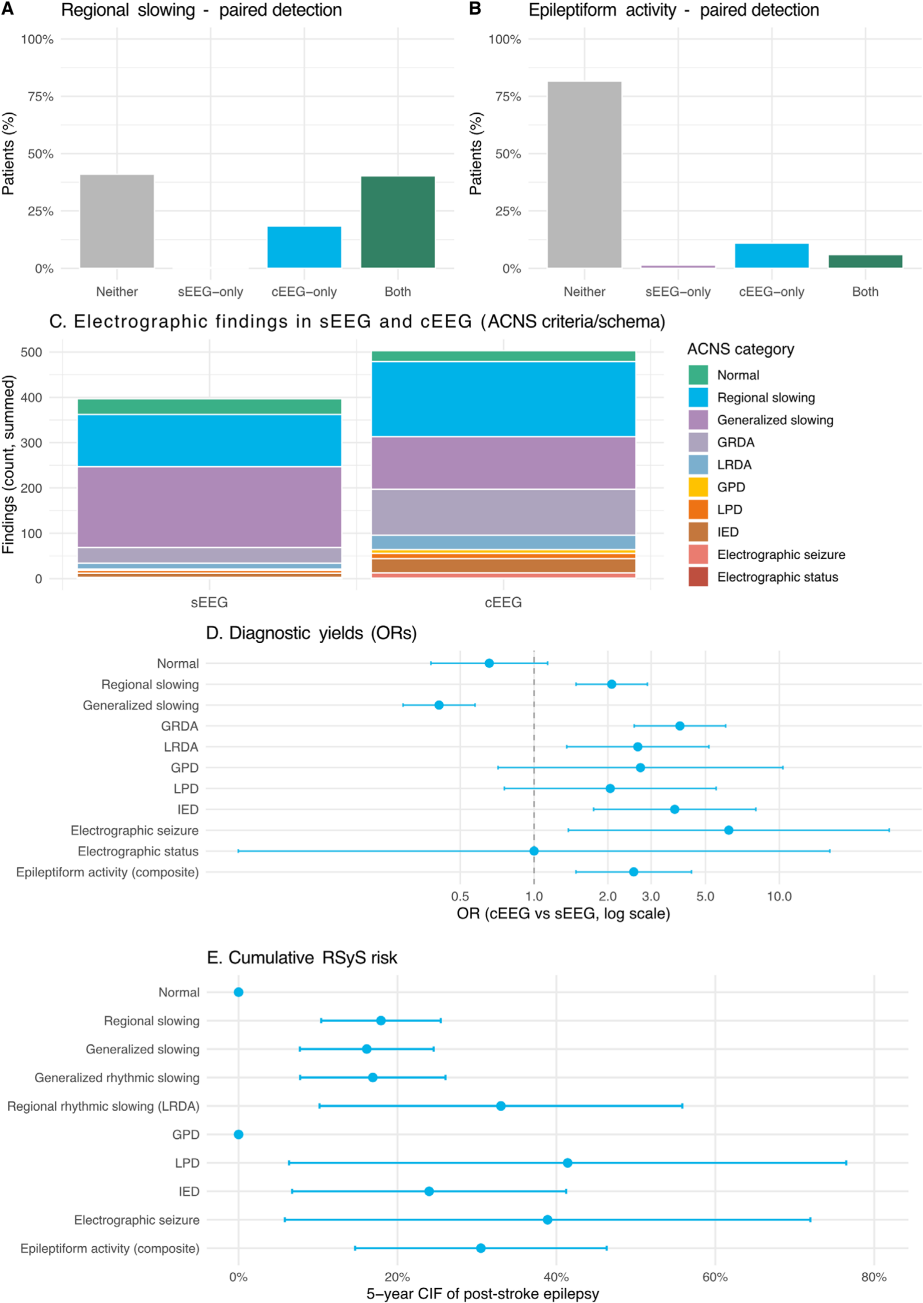
Diagnostic yield of sEEG versus cEEG and association with PSE by ACNS categories. Panels (A, B) show paired detection within subjects for regional slowing A and EA (composite of IED, LPD, GPD, and electrographic seizure/status) B, partitioned as neither, sEEG‐only, cEEG‐only, or both. Panel (C) summarizes the count of ACNS findings by modality (*stacked bars*; identical ordering across modalities). Panel (D) displays ORs (log scale) with 95% CIs for detection on cEEG versus sEEG for each ACNS category (*vertical dashed line* at OR = 1). Panel (E) shows the cumulative PSE risk (point estimate with Wilson 95% CI) among patients with a given cEEG finding. The cEEG preferentially detected pathologic features – most prominently GRDA, LRDA, IEDs, and electrographic seizures – whereas generalized slowing was more often seen on sEEG. Findings with higher detection on cEEG (eg, LPD, seizures, and the EA composite) corresponded to higher cumulative PSE risk on cEEG. ACNS = American Clinical Neurophysiology Society; cEEG = continuous electroencephalography; CI = confidence interval; CIF = cumulative incidence function; EA = epileptiform activity; GPD = generalized periodic discharge; GRDA = generalized rhythmic delta activity; IED = interictal epileptiform discharge; LPD = lateralized periodic discharge; LRDA = lateralized rhythmic delta activity; OR = odds ratio; PSE = post‐stroke epilepsy; SE = status epilepticus; sEEG = short electroencephalography. [Color figure can be viewed at www.annalsofneurology.org]

The cEEG detected substantially more abnormalities than sEEG (Fig 1A–D, and Table 2). Compared to sEEG, cEEG significantly increased detection of regional slowing (59 vs 41%, OR = 2.1, 95% CI = 1.5–2.9, *p* < 0.001), GRDA (36 vs 12%, OR = 3.9, 95% CI = 2.6–6.0, *p* < 0.001), LRDA (11 vs 5%, OR = 2.7, 95% CI = 1.4–5.3, *p* = 0.004), IEDs (11 vs 3%, OR = 3.8, 95% CI = 1.8–8.0, *p* < 0.001), and electrographic seizures (4 vs 1%, OR = 6.2, 95% CI = 1.4–28.1, *p* = 0.01). EA almost always co‐occurred with slowing. Among the abnormalities included in the SeLECT‐EEG score, any EA increased from 18 of 283 (6%) on sEEG to 66 of 283 (23%) on cEEG, and regional slowing rose from 115 of 283 (41%) to 166 of 283 (59%; see Fig 1A, B).

**TABLE 1:** Fine–Gray sHRs by PSE

Variables	Contrast/Level or Comment	sHR (95% CI)	*p*
Age, yr – median (IQR)	≥64 vs <64	1.20 (0.66–2.19)	0.55
Sex – F	F vs M	0.93 (0.51–1.70)	0.81
NIHSS at admission – ≤3	≤3 vs >3	0.60 (0.29–1.23)	0.17
NIHSS at admission – 4–10	4–10 vs not 4–10	0.48 (0.20–1.14)	0.10
NIHSS at admission – ≥11	≥11 vs <11	2.43 (1.30–4.54)	**0.006**
MCA territory involvement	Present vs absent	2.73 (1.09–6.79)	**0.03**
Cortical involvement	Present vs absent	1.66 (0.81–3.39)	0.16
Large‐artery atherosclerosis	Present vs absent	1.60 (0.84–3.03)	0.15
All other causes	≥64 vs <64	1.20 (0.66–2.19)	0.55
EEG pattern on cEEG – ACNS schema			
Normal EEG	0 cases in PSE	‐	‐
Generalized slowing	Present vs absent	1.04 (0.56–1.93)	0.90
Regional slowing	Present vs absent	1.92 (0.95–3.89)	0.07
GRDA	Present vs absent	1.06 (0.57–1.98)	0.85
LRDA	Present vs absent	1.96 (0.91–4.23)	0.09
GPDs	Present vs absent	0.74 (0.11–4.97)	0.76
LPDs	Present vs absent	4.50 (2.13–9.51)	**< 0.001**
IEDs	Present vs absent	2.14 (1.00–4.55)	**0.05**
Electrographic seizure	Present vs absent	3.63 (1.52–8.63)	**0.004**
Electrographic SE	0 cases in PSE	‐	‐

*Note*: Prespecified variable from Supplementary Table S1 was evaluated in a separate Fine–Gray model for time to PSE with death as the competing event; results are reported as sHRs with 95% CI and Wald *p* value. Age was dichotomized at the cohort median (≥64 vs <64), NIHSS was represented by 3 binary contrasts (≤3 vs >3, 4–10 vs not 4–10, and ≥11 vs <11), and imaging/EEG variables were modeled as present vs absent. Higher stroke severity was strongly associated with PSE (NIHSS ≥ 11 vs < 11: sHR = 2.43, 95% CI = 1.30–4.54, *p* = 0.006), as was MCA territory involvement (sHR = 2.73, 95% CI = 1.09–6.79, *p* = 0.03). Among EEG features, LPD (sHR = 4.50, 95% CI = 2.13–9.51, *p* < 0.001) and electrographic seizures (sHR = 3.63, 95% CI = 1.52–8.63, *p* = 0.004); IED met nominal significance (sHR = 2.14, 95% CI = 1.00–4.55, *p* = 0.05), whereas LRDA and regional slowing trended positively with wider intervals (LRDA sHR = 1.96, 95% CI = 0.91–4.23, *p* = 0.09; regional slowing sHR = 1.92, 95% CI = 0.95–3.89, *p* = 0.07). Generalized slowing (sHR = 1.04, 95% CI = 0.56–1.93, *p* = 0.90), GRDA (sHR = 1.06, 95% CI = 0.57–1.98, *p* = 0.85), and age ≥ 64 (sHR = 1.20, 95% CI = 0.66–2.19, *p* = 0.55) were not significant. Rows with 0 PSE cases in the “Present” group are shown for completeness and are non‐estimable. Bold values indicate statistical significance, defined as two‐sided p < 0.05. Exact *p* values are reported unless *p* < 0.001.

ACNS = American Clinical Neurophysiology Society; cEEG = continuous electroencephalography; CI = confidence interval; EEG = electroencephalography; GPDs = generalized periodic discharges; GRDA = generalized rhythmic delta activity; HRs = hazard ratios; IEDs = interictal epileptiform discharges; IQR = interquartile range; LPDs = lateralized periodic discharges; LRDA = lateralized rhythmic delta activity; MCA = middle cerebral artery; NIHSS = National Institutes of Health Stroke Scale; SE = status epilepticus; sHR = subdistribution hazard ratio.

**TABLE 2:** Detection of EEG Abnormalities on sEEG Versus cEEG (n = 283)

Variables	sEEG, n (%)	cEEG, n (%)	OR (95% CI) cEEG vs sEEG	*p*
EEG pattern on cEEG — ACNS schema				
Normal	35 (12.4)	24 (8.5)	0.66 (0.38–1.14)	0.17
Regional slowing	115 (40.6)	166 (58.7)	2.07 (1.48–2.90)	**< 0.001**
Generalized slowing	178 (62.9)	116 (41.0)	0.41 (0.29–0.57)	**< 0.001**
GRDA	35 (12.4)	101 (35.7)	3.93 (2.56–6.04)	**< 0.001**
LRDA	13 (4.6)	32 (11.3)	2.65 (1.36–5.16)	**< 0.001**
GPDs	3 (1.1)	8 (2.8)	2.72 (0.71–10.34)	0.22
LPDs	6 (2.1)	12 (4.2)	2.04 (0.76–5.52)	0.23
IEDs	9 (3.2)	31 (11.0)	3.75 (1.75–8.02)	**< 0.001**
Electrographic seizure	2 (0.7)	12 (4.2)	6.22 (1.38–28.06)	**0.01**
Electrographic SE	1 (0.4)	1 (0.4)	1.00 (0.06–16.07)	1.00

*Note*: Detection of EEG abnormalities on sEEG versus cEEG in the full cohort (n = 283). Entries show counts (%) detected on each modality, with ORs comparing cEEG to sEEG and 2‐sided *p* values. Compared with sEEG, cEEG identified more regional slowing (40.6 vs 58.7%, OR = 2.07, 95% CI = 1.48–2.90, *p* < 0.001), GRDA (12.4 vs 35.7%, OR = 3.93, 95% CI = 2.56–6.04, *p* < 0.001), LRDA (4.6 vs 11.3%, OR = 2.65, 95% CI = 1.36–5.16, *p* < 0.001), IEDs (3.2 vs 11.0%, OR = 3.75, 95% CI = 1.75–8.02, *p* < 0.001), and electrographic seizures (0.7 vs 4.2%, OR = 6.22, 95% CI = 1.38–28.06, *p* = 0.01); generalized slowing was more frequent on sEEG (62.9 vs 41.0%, OR = 0.41, 95% CI = 0.29–0.57, *p* < 0.001). Bold values indicate statistical significance, defined as two‐sided *p* < 0.05. Exact p values are reported unless *p* < 0.001.

ACNS = American Clinical Neurophysiology Society; cEEG = continuous electroencephalography; CI = confidence interval; EEG = electroencephalography; GPDs = generalized periodic discharges; GRDA = generalized rhythmic delta activity; IEDs = interictal epileptiform discharges; LPDs = lateralized periodic discharges; LRDA = lateralized rhythmic delta activity; OR = odds ratio; SE = status epilepticus; sEEG = short electroencephalography.

### Association of EEG Abnormalities with Post‐Stroke Epilepsy

**FIGURE 2:**
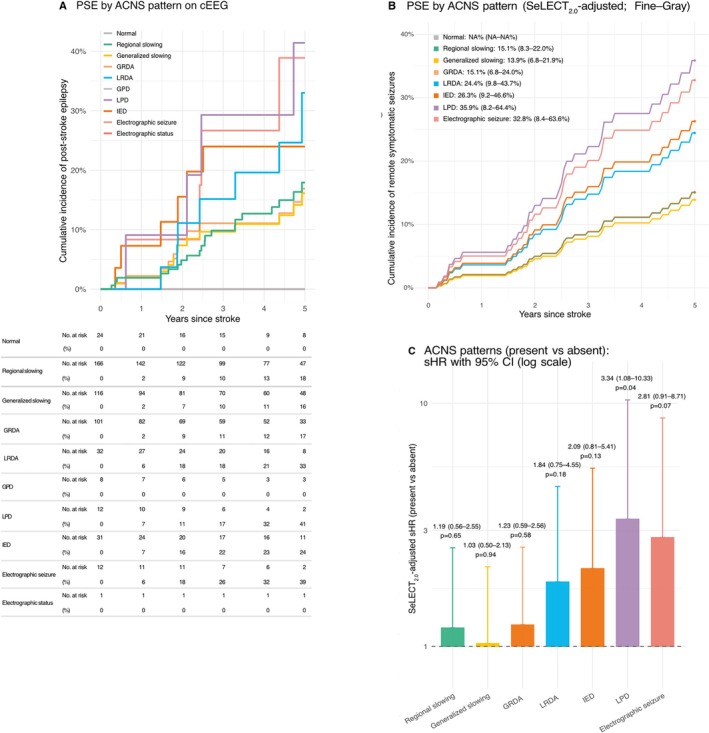
RSS risk by ACNS EEG finding. Panel (A) shows unadjusted CIFs for RSS within 5 years, stratified by the presence of individual ACNS patterns on cEEG (non‐exclusive; death treated as a competing event). Epileptiform abnormalities carried the highest risk: 5‐year CIFs were 39% for electrographic seizures, 41% for LPD, 33% for LRDA, and 24% for IED. Slowing patterns were lower: generalized slowing 16%, regional slowing 18%, and GRDA 17%. Patients with a normal cEEG did not develop PSE within 5 years. Panel (B) shows SeLECT_2.0_‐adjusted CIFs estimated from Fine–Gray regression fit separately for each pattern (presence vs absence) with SeLECT_2.0_ included as a covariate; curves are displayed for the pattern‐present subgroup with SeLECT_2.0_ fixed at that pattern's mean. Adjustment preserved the ranking and shifted epileptiform patterns upward, while slowing patterns remained associated with lower incidence. Panel (C) displays SeLECT_2.0_‐adjusted sHRs from the same Fine–Gray models, reported as pattern present versus absent. The highest adjusted hazards were observed for LPD (sHR = 3.34), electrographic seizure (sHR = 2.81), IED (sHR = 2.09), whereas regional (sHR = 1.19) and generalized slowing (sHR = 1.03) showed lower hazards; such as SE. ACNS = American Clinical Neurophysiology Society; cEEG = continuous electroencephalography; CIF = cumulative incidence function; GPD = generalized periodic discharge; GRDA = generalized rhythmic delta activity; IED = interictal epileptiform discharge; LPD = lateralized periodic discharge; LRDA = lateralized rhythmic delta activity; PSE = post‐stroke epilepsy; SE = status epilepticus; sHR = subdistribution hazard ratio. [Color figure can be viewed at www.annalsofneurology.org]

Abnormalities detected on cEEG were strongly associated with PSE (Fig 1E and 2, Table 1, and Supplementary Fig S2). Unadjusted Fine–Gray models identified LPDs (sHR = 4.5, 95% CI = 2.1–9.5), electrographic seizures (sHR = 3.6, 95% CI = 1.5–8.6), and IEDs (sHR = 2.1, 95% CI = 1.0–4.6) as the strongest predictors of PSE; LRDA (sHR = 2.0, 95% CI = 0.9–4.2) and regional slowing (sHR = 1.9, 95% CI = 1.0–3.9) trended positive, whereas generalized slowing and GRDA were not associated with excess risk (see Table 1). For SeLECT_2.0_‐adjusted estimated risk of PSE at 5 years (see Fig 2B, C), these patterns showed the same ranking: LPDs, electrographic seizures, IEDs, and LRDA—36% (8–64), 33% (8–64), 26% (9–47), and 24% (10–44) with sHR = 3.3 (95% CI = 1.1–10.3, *p* = 0.04), sHR = 2.8 (95% CI = 0.9–8.7, *p* = 0.07), sHR = 2.1 (95% CI = 0.8–5.4, *p* = 0.13), and sHR = 1.8 (95% CI = 0.8–4.6, *p* = 0.19), respectively; regional slowing and GRDA were lower (15% [8, 9, 10, 11, 12, 13, 14, 15, 16, 17, 18, 19, 20, 21, 22] and 14–15%) with sHR = 1.2 (95% CI = 0.6–2.6) and sHR = 1.0 (95% CI = 0.5–2.1), whereas normal EEG (n = 24), GPDs (n = 8), and electrographic SE epilepticus (n = 1) had no PSE cases.

For the 2 SeLECT‐EEG targets (EA and regional slowing), cEEG‐only detection carried higher 5‐year risk than “pattern never present”: EA 28 versus 11% (Gray *p* = 0.006, adjusted *p* value [*p*_adj] = 0.04), and regional slowing 26 versus 13% (Gray *p* = 0.02, *p*_adj = 0.18), the latter not statistically significant after correction (see Supplementary Fig S2).

**FIGURE 3:**
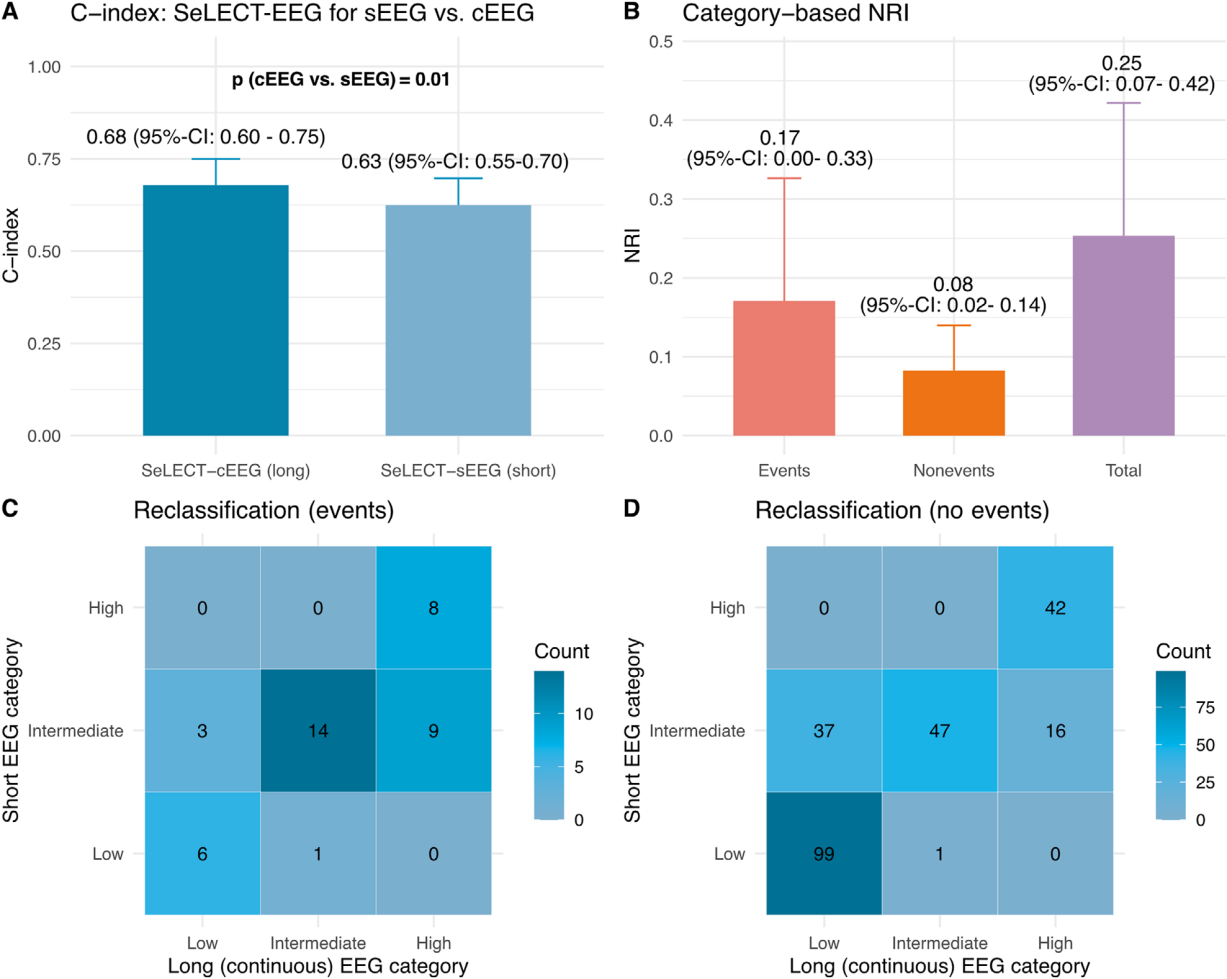
Prognostic performance of sEEG versus cEEG for predicting post‐stroke epilepsy. Figure 3 summarizes model discrimination, reclassification, and reclassification structure when SeLECT‐EEG scores are computed from sEEG (60 minutes) versus cEEG (>12 hours). Panel (A) displays Harrell's concordance index for the short‐based and continuous‐based scores as vertical bars, with upward error bars showing bootstrap 95% CIs (1,000 replicates) and on‐bar labels showing the point estimates; the 2‐sided bootstrap *p* value for the difference in concordance (continuous vs short) is annotated above the bars. Panel (B) presents category‐based net reclassification improvement using tertiles of predicted risk (low, intermediate, and high) with separate bars for events, nonevents, and the total; bars show point estimates with upward 95% CIs from 1,000 bootstrap replicates. Panels (C) and (D) show the reclassification contingency tables as heatmaps with cell counts overlaid: the horizontal axis gives the 3 risk categories from the cEEG‐based score, the vertical axis gives the 3 risk categories from the sEEG‐based score, and the color scale reflects cell frequencies; each tile is labeled with the observed count to facilitate inspection of upward and downward movement across categories. Together, the 4 panels visualize that incorporating findings from cEEG improves discrimination and yields clinically meaningful upward movement among patients who developed post‐stroke epilepsy while appropriately down‐classifying many without seizures. cEEG = continuous electroencephalography; CI = confidence interval; sEEG = short electroencephalography. [Color figure can be viewed at www.annalsofneurology.org]

Adding EEG markers improved prognostic performance: SeLECT‐EEG based on cEEG achieved a C‐index 0.68 (95% CI = 0.61–0.75) versus 0.63 (95% CI = 0.55–0.70) with sEEG (ΔC = 0.055, 95% CI = 0.012–0.101, *p* = 0.014). Category‐based NRI across SeLECT‐EEG tertiles showed event‐NRI +0.17 (ie, improved upward reclassification among patients who developed PSE) and non‐event‐NRI +0.08 (improved downward reclassification among those who did not develop PSE), for a total NRI +0.25 (95% CI = 0.07–0.42; Fig 3).

### sEEG Finding Implications: When Does Extending to cEEG Add Value for Predicting Early Electrographic Seizures/SE and Epileptiform Activity?

We assessed the performance of the clinical prescreener. In the larger SeLECT cohort (n = 4,552), Supplementary Table S1 showed that a SeLECT_2.0_ cutoff of ≥4 maximized the Youden index (0.33), balancing sensitivity and specificity for identifying future PSE. This threshold therefore defined the clinical high‐risk stratum for sEEG finding analyses.

**FIGURE 4:**
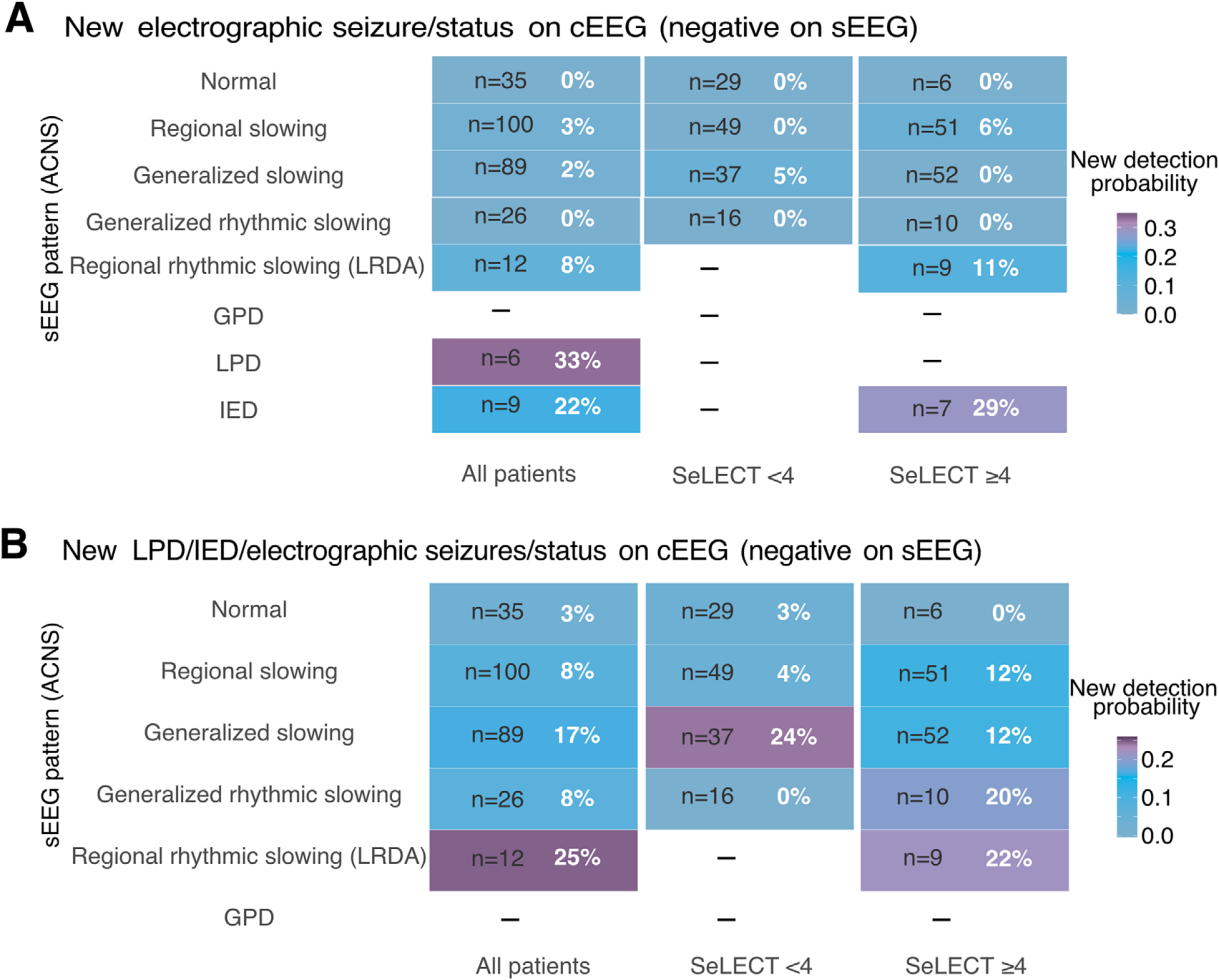
Pattern heatmaps for new electrographic activity on cEEG. Rows show sEEG patterns using ACNS terminology; columns stratify SeLECT_2.0_ (all, <4, ≥4). Panel (A) displays the probability that cEEG newly detects electrographic seizures or status among patients who were sEEG‐negative for electrographic seizures/status. Panel (B) displays the probability that cEEG newly detects IED/LPD/electrographic seizures/status among patients sEEG‐negative for that composite. Tiles report the cell percentage and n; cells with n < 5 are muted. Probabilities use Wilson binomial estimates. “All patients” equals the sum of the SeLECT subcolumns. In panel A, new detection concentrates in epileptiform‐leaning rows, with the highest yields in LPD (2/6, 33%) and IED (2/9, 22%) albeit with small n; among rows with n ≥ 5, LRDA is approximately 8% overall (1/12; 11% in SeLECT ≥ 4), whereas regional slowing and generalized slowing remain low (≈3% [3/100] and 2% [2/89]); in SeLECT < 4, the only non‐zero cell with n ≥ 5 is generalized slowing (5.4%, 2/37). In panel B, yields are higher: LRDA 25% (3/12; 22% in SeLECT ≥ 4), generalized slowing 17% overall (24% in < 4; 11.5% in ≥ 4), regional slowing 8% overall (11.8% in ≥ 4), GRDA 7.7% overall (20% in ≥ 4), whereas normal is low (2.9%, 1/35). ACNS = American Clinical Neurophysiology Society; cEEG = continuous electroencephalography; GRDA = generalized rhythmic delta activity; IED = interictal epileptiform discharge; LPD = lateralized periodic discharge; LRDA = lateralized rhythmic delta activity (regional rhythmic slowing); sEEG = short electroencephalography; SeLECT = always SeLECT_2.0_ used. [Color figure can be viewed at www.annalsofneurology.org]

In the EEG pattern heatmaps (Fig 4A; outcome = electrographic seizures/SE), overall cEEG yield after an electrographic seizures/SE negative sEEG were highest for LPDs (33%) and IEDs (22%), followed by LRDA (8%), whereas regional slowing (3%) and generalized slowing (2%) were low; normal remained low as well. For the broader composite (IEDs/LPDs/ electrographic seizures/SE; Fig 4B), overall yields were LRDA 25%, GRDA 8%, generalized slowing 17%, regional slowing 8%, and normal 3%. The accompanying points‐based models (derived from multivariable logistic regression using the same early EEG predictors as the heatmap strata) supported a heatmap‐first strategy (Supplementary Fig S4): the electrographic seizures/SE model showed good discrimination (cross‐validated‐median AUC 0.71) with acceptable calibration, whereas the IED/LPD/electrographic seizures/SE model had modest discrimination (cross‐validated‐median AUC approximately 0.63); decision‐curve and precision‐recall performance were modest for both, supporting use of the heatmaps as the primary prioritization tool and the scores for within‐cell ranking rather than hard point cutoffs (see Supplementary Fig S4).

### Future Prioritization Rule (Predicting PSE Only) when Does Extending cEEG Add Value for Prediction of Later PSE?

**FIGURE 5:**
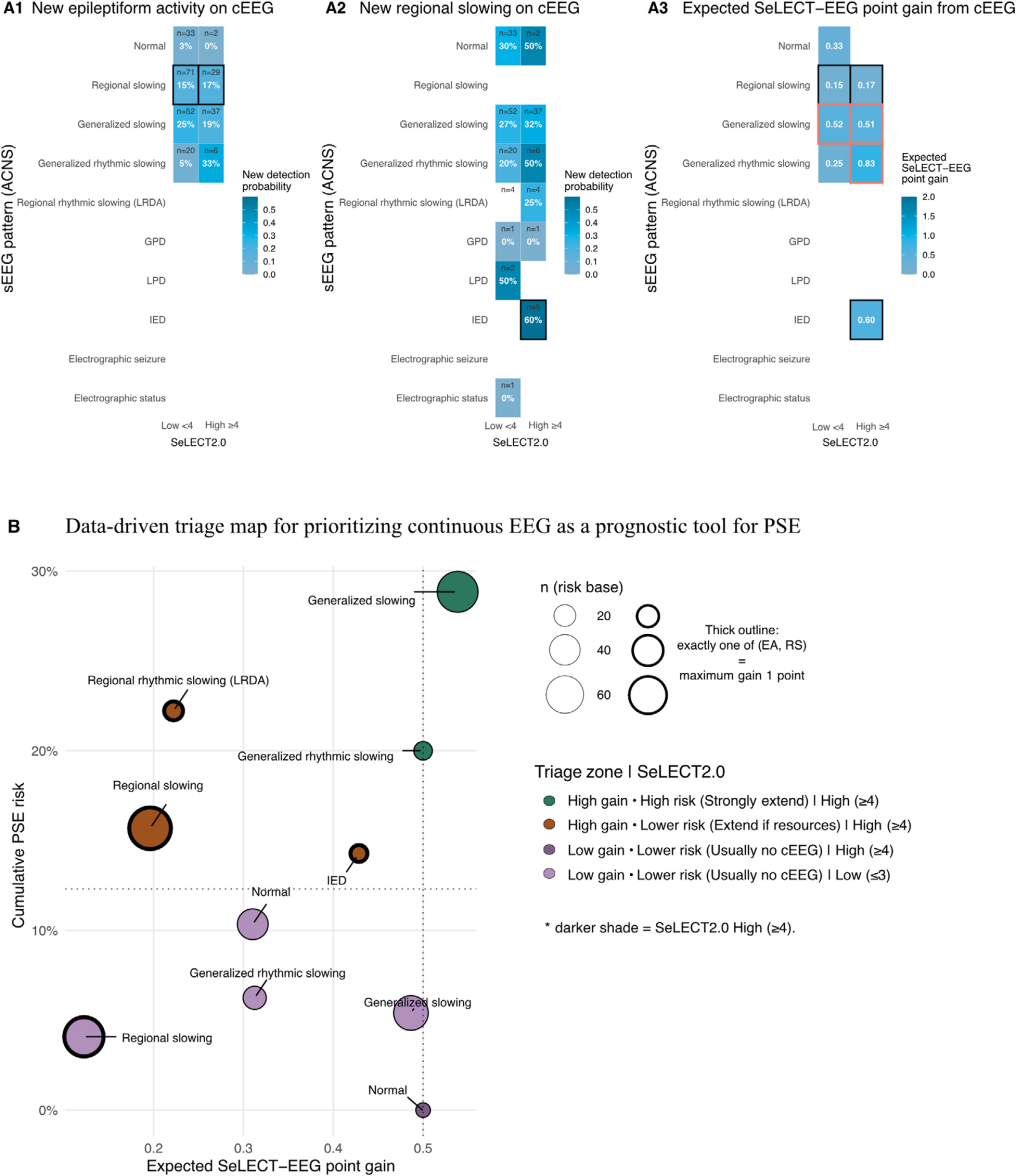
Data‐driven pattern map for prioritizing continuous EEG as a prognostic tool for RSS. Panel (A1) shows, by sEEG row and SeLECT 2.0 column (low < 4 and high ≥ 4), the probability that cEEG newly detects epileptiform activity (EA) when sEEG was EA–. Panel (A2) analogously shows new regional slowing when sEEG was regional slowing negative. Panel (A3) encodes the expected SeLECT‐EEG point gain from adding cEEG as P (new EA) + P (new regional slowing), dimming cells where both EA and regional slowing were already present on sEEG (no additional gain possible) and framing high‐gain/high‐support cells (expected gain > 0.5 points and n_support > 5). Heatmap tiles display the cell percentage and n using Wilson estimates; cells with n < 5 are muted and the color scale is anchored at 0 and capped near each panel's observed maximum. Across A1 through A3, higher yields and larger expected gains cluster in slowing and epileptiform‐leaning rows (eg, LRDA, generalized/ regional slowing), whereas normal and SeLECT < 4 cells remain low‐yield. Panel (B) integrates these findings into a prioritization map: each bubble (pattern × SeLECT cell) plots expected point gain (x‐axis) against cumulative PSE risk (y‐axis); bubble size scales with n, thick outlines mark cells with exactly one of regional slowing or EA finding on sEEG, and white fill indicates both already present (no added points). Dashed lines mark the prespecified gain threshold (0.5 points) and the median PSE risk, defining 4 practical zones—high‐gain•high‐risk (strongly extend cEEG), high‐gain•lower‐risk (extend if resources), low‐gain•high‐risk (targeted cEEG), and low‐gain•lower‐risk (usually no cEEG). In this space, SeLECT ≥ 4 cells predominantly occupy the higher‐risk half‐plane (effective pre‐screen), and the largest expected gains concentrate in isolated slowing phenotypes, with generalized slowing consistently exceeding isolated regional slowing. cEEG = continuous electroencephalography; EA = epileptiform activity; Electrographic status = electrographic status epilepticus; IED = interictal epileptiform discharge; LPD = lateralized periodic discharge; LRDA = lateralized rhythmic delta activity; PSE = post‐stroke epilepsy; sEEG = short electroencephalography; SeLECT = always SeLECT_2.0_ used. [Color figure can be viewed at www.annalsofneurology.org]

Using the prespecified SeLECT_2.0_ ≥ 4 cutoff point (SeLECT‐high), detection patterns and expected SeLECT‐EEG gain concentrated in predictable EEG pattern groups: high‐gain tiles clustered around sEEG phenotypes dominated by slowing with generalized slowing showing larger expected gains (≈0.5–0.8 points) than isolated regional slowing (≈0.3–0.5). SeLECT‐high cells had higher incremental yield, with mean expected gain ≈0.10 points and ≈0.07 points more per possible point than SeLECT‐low (both *p* < 0.001; Supplementary Table S3). In the bubble map (Fig 5), SeLECT‐high with generalized slowing populated the high‐risk/high‐gain quadrant, SeLECT‐high with regional slowing lay in high‐risk but lower‐gain, and SeLECT‐low patterns consistently occupied low‐risk/low‐gain. Applying this prognostic framework to the larger SeLECT cohort (n = 4,552; excluding ASyS n = 4,319) yielded SeLECT ≥ 4 in 33% (approximately 1,425 patients). Based on our prioritization definitions, expanded criteria (high risk with either high or modest expected gain) would flag 30% of 4,319 (approximately 1,296) for cEEG, whereas a stricter rule requiring both high risk and high gain (≥ 0.5 points) would include 15% (approximately 648; Supplementary Fig S5).

## Discussion

Early EEG is among the few accessible biomarkers of cortical dysfunction and hyperexcitability in the acute post‐stroke period. This is particularly relevant because ASyS, the clinical manifestation of cortical hyperexcitability, occur in only a minority of ischemic strokes. Yet, PSE remains a major source of acquired epilepsy burden.^1^, ^2^, ^3^ In this multicenter cohort of 283 ischemic stroke survivors without ASyS who underwent cEEG within 7 days, we quantified what prolonged monitoring adds beyond a 60‐minute sEEG by defining sEEG as the first hour of the same recording. With sampling duration as the main difference between modalities, cEEG (i) increased detection of epileptiform and rhythmic/periodic abnormalities, (ii) captured “late” abnormalities that improved PSE risk stratification, and (iii) supported phenotype‐ and risk‐guided decisions about when to extend monitoring.

### Added Diagnostic Yield of cEEG

Extending monitoring beyond the first hour increased detection of several clinically meaningful patterns by approximately 2‐ to 4‐fold, including regional slowing, rhythmic/periodic activity, epileptiform discharges, and electrographic seizures. This duration dependence aligns with critical care EEG evidence that nonconvulsive seizures and rhythmic/periodic patterns are frequently intermittent and accrue over time,^17^, ^18^, ^19^, ^20^, ^21^ and with consensus statements emphasizing improved detection of nonconvulsive seizures and periodic/rhythmic discharges in appropriately selected high‐risk patients.^22^, ^23^, ^24^ Stroke‐specific cEEG studies similarly show that clinically occult epileptic activity is not rare (electrical epileptic activity approximately 17% and electrographic seizures approximately 2% in a prospective stroke‐unit cohort).^25^ The detection rates for electrographic seizures and rhythmic/periodic patterns vary substantially across contemporary series (up to approximately 12% and approximately 25%, respectively), mainly reflecting differences in cohort selection and EEG strategy.^7^, ^10^, ^11^, ^25^, ^26^

A key strength of this within recording comparison is that sEEG and cEEG differed only by sampling duration. Generalized slowing was relatively more prominent in the first hour, whereas focal and rhythmic or periodic abnormalities were detected more often with longer monitoring, consistent with resolution of early diffuse encephalopathy and improved capture of intermittent cortical hyperexcitability. Analogous time‐dependent evolution has been emphasized in post‐anoxic coma and disorders of consciousness cohorts.^27^, ^28^ In stroke cohorts, serial short EEGs similarly increase detection of epileptiform phenomena compared with the first study alone.^26^ Identifying these later abnormalities is prognostically relevant given their association with subsequent PSE.^7^, ^10^, ^11^, ^26^

### Prognostic Implications for PSE

The cEEG abnormalities stratified PSE risk across a marked gradient. The highest risks were observed with electrographic seizures and lateralized periodic discharges, intermediate risks with lateralized rhythmic delta activity, and interictal epileptiform discharges, and lower risks with slowing‐only phenotypes. Notably, patients with a normal cEEG had no PSE events during follow‐up, suggesting a potentially informative rule‐out signal that warrants confirmation in broader, less selected stroke‐unit populations. These gradients are consistent with prior stroke‐EEG studies and with acute brain‐injury data linking rhythmic/periodic activity to physiologic stress and adverse outcomes in a burden‐dependent manner.^7^, ^10^, ^11^, ^26^, ^29^, ^30^, ^31^, ^32^

Beyond association, prolonged sampling improved prognostic performance when SeLECT‐EEG was computed from cEEG rather than from the first hour alone, producing a modest but statistically significant improvement in discrimination and risk reclassification. This supports the interpretation that longer monitoring adds clinically relevant prognostic information rather than merely increasing detection.

### When to Extend Beyond the First Hour: Acute Detection and Prognosis‐Oriented Prioritization

When the initial hour is normal, subsequent electrographic seizures are uncommon, and the emergence of clinically actionable epileptiform/rhythmic findings is low. In contrast, early abnormalities—particularly focal or rhythmic/periodic patterns—identify patients in whom extended monitoring more often reveals additional epileptic activity and/or seizures. This mirrors the broader cEEG literature: absence of early epileptiform/rhythmic features predicts low subsequent seizure yield, whereas early epileptiform or rhythmic patterns justify extension because events are frequently nonconvulsive and time‐dependent.^17^, ^18^, ^19^, ^20^, ^21^ Our approach complements general inpatient seizure‐risk tools such as 2HELPS2B by tailoring decisions to a single, high‐prevalence etiology and incorporating stroke‐specific clinical prior risk (SeLECT_2.0_).^14^ Prior multicenter work further suggests that electrographic seizures may confer long‐term seizure risk comparable to clinically observed acute seizures, reinforcing the value of capturing covert electrographic events even when clinical ASyS are absent.^7^

#### Prognosis‐Oriented Yield

**FIGURE 6:**
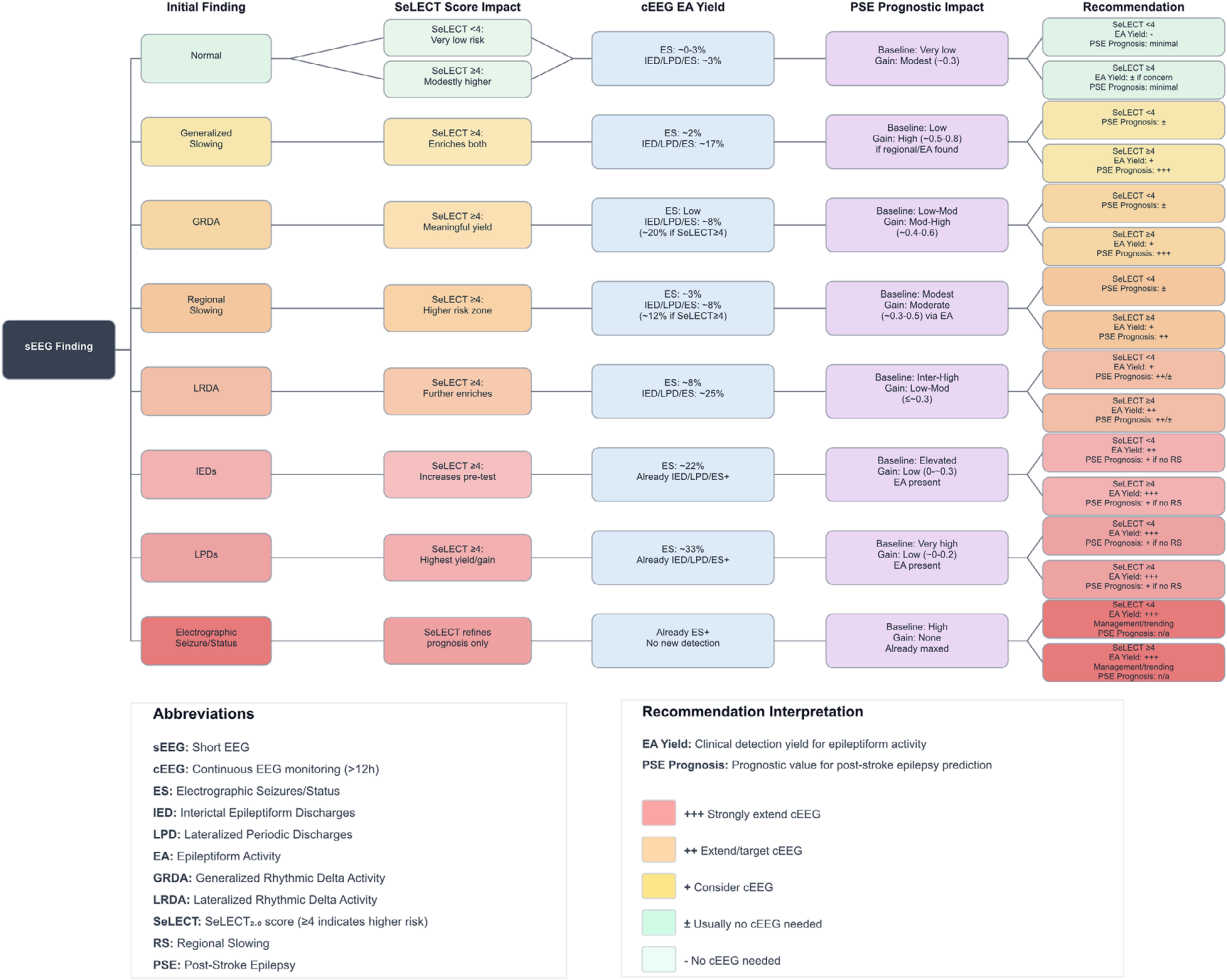
The sEEG‐guided prioritization for cEEG: epileptiform abnormality yield and incremental PSE prognostic value by SeLECT_2.0_ stratum. This figure summarizes how the initial sEEG findings and SeLECT_2.0_ stratum (<4 vs ≥4) can be used to prioritize performing cEEG for 2 purposes: (i) increasing yield of EA not seen on the first hour, and (ii) PSE prognostication risk refinement via incremental SeLECT‐EEG information gained when cEEG newly identifies regional slowing and/or epileptiform activity. “EA detection yield” refers to the probability that cEEG newly detects electrographic seizures/status or the broader composite of epileptiform findings among patients who were negative for that end point on sEEG; when epileptiform activity is already present on sEEG, the composite yield is not applicable and cEEG primarily serves to quantify burden, capture seizures/status, and guide monitoring/management rather than to add new prognostic points. Prognostic “gain” is expected to be greatest when sEEG shows nonspecific abnormalities that may upgrade on cEEG (eg, generalized slowing evolving to regional slowing and/or epileptiform activity), particularly in the SeLECT ≥ 4 stratum, and lowest when sEEG already demonstrates epileptiform activity or electrographic seizures/status. Recommendation symbols indicate strength of support for extending monitoring in a resource‐aware framework (cEEG EA yield and cEEG PSE prognostic impact indications may diverge). More details in Supplementary Table S4. cEEG = continuous electroencephalography; EA = epileptiform activity; PSE = post‐stroke epilepsy; sEEG = short electroencephalography; SeLECT = always SeLECT_2.0_ used. [Color figure can be viewed at www.annalsofneurology.org]

In patients without ASyS, extended EEG can be pursued for improved long‐term risk classification. This currently has limited immediate therapeutic consequence because no antiepileptogenic therapy is established and routine prophylactic antiseizure medication is not recommended.^2^, ^3^ However, prognosis‐oriented stratification is increasingly relevant because epileptogenesis and thus seizure risk is temporally clustered and observational and repurposing analyses suggest potentially modifiable epilepsy outcomes if confirmed prospectively.^33^, ^34^, ^35^, ^36^, ^37^, ^38^, ^39^ We operationalized prognostic prioritization by combining baseline PSE risk (SeLECT 2.0) with the expected SeLECT‐EEG point gain from extending sEEG to cEEG. Patients with SeLECT_2.0_ ≥ 4 concentrated the highest expected gains. In this high‐risk stratum, extension after isolated generalized slowing yielded the largest expected score increases (≈0.5–0.8 SeLECT‐EEG points), whereas isolated regional slowing produced smaller but still clinically relevant gains (≈0.3–0.5 points). Across patterns, expected gain was consistently higher in SeLECT_2.0 _≥ 4 than in SeLECT_2.0_ < 4 (about +0.10 points overall and +0.07 points per available point, *p* < 0.001; see Supplementary Table S3). Two implications follow. First, prognostic yield is maximized when sEEG shows diffuse/nonspecific abnormalities (especially generalized slowing) and clinical prescreening indicates higher baseline risk; this is where cEEG most often upgrades patients by uncovering epileptiform activity or regional slowing that changes SeLECT‐EEG classification.^17^, ^18^, ^19^, ^20^, ^21^ This effect is further enriched when prescreening identifies higher baseline clinical risk where expected point gain and reclassification are concentrated. Second, when clearly high‐risk markers are already present on sEEG, additional monitoring adds less incremental prognostic information (risk is already established), but can still be clinically relevant to detect electrographic seizures/SE and quantify burden, which may influence acute management. A pragmatic summary of prioritization implications is provided in Figure 6 and Supplementary Table S4.

#### Feasibility and Scale

In the broader SeLECT registry, after excluding ASyS, approximately one third of patients have SeLECT_2.0_ ≥ 4; within this higher‐risk stratum, approximately 30% meet an “expanded” prioritization definition and 15% meet a stricter high‐risk/high‐gain definition (see Fig 5 and Supplementary Fig S5). These proportions suggest that prognosis‐oriented prioritization would select a sizable subset of patients without acute symptomatic seizures for extended monitoring, without implying universal cEEG. Increasing availability of point of care EEG and artificial intelligence (AI) supported review may enable scalable deployment within stepwise pathways linking stroke units to first seizure services.^40^, ^41^, ^42^, ^43^, ^44^, ^45^ Clinical impact will depend on pairing stratification with predefined downstream care, because first seizure clinic models have been associated with fewer subsequent emergency presentations and admissions and early treatment strategies in new onset epilepsy may influence recurrence.^46^, ^47^ These considerations support prospective multicenter stroke unit studies that integrate standardized early EEG phenotyping with structured follow‐up pathways and risk enrichment for prevention or drug repurposing trials.^48^, ^49^

### Limitations and Future Directions

Several limitations should calibrate inference. First, this was a retrospective, clinically indicated cEEG cohort assembled from 2 subcohorts with different indications for EEG monitoring and workflows. Therefore, confounding by indication and enrichment for higher risk patients limit generalizability. Such real‐world heterogeneity is a strength (capturing different workflows) but also means that absolute detection rates in the pattern heatmaps may be center‐dependent; accordingly, we present them as exploratory prioritization aids when cEEG capacity is constrained. Second, the within recording design isolates duration effects but does not fully emulate routine clinical workflows in which routine EEG is performed separately and cEEG may be initiated later under different state and treatment conditions. Third, we did not model the time from stroke onset to cEEG initiation. In retrospective chart review, accurate extraction of stroke‐to‐EEG latency can be uncertain (eg, imprecise onset times, inter‐hospital transfers, and documentation variability). Nonetheless, our results are intended to apply to patients who undergo EEG monitoring at any time within the first 7 days after ischemic stroke. Fourth, some patterns and end points were infrequent, limiting precision for subgroup estimates and favoring interpretation of phenotype‐based gradients over rigid cutoff points. Fifth, treatment is a potential time‐dependent confounder because antiseizure medication or sedation may be initiated in response to EEG findings. Sixth, we excluded patients with acute symptomatic seizures by design; therefore, our findings are not intended to guide EEG versus cEEG prioritization in ASyS‐positive patients. This is a clinically important group in whom some events labeled as ASyS may represent seizure mimics or uncertain semiologies, and EEG/cEEG could, in principle, contribute to diagnostic adjudication and potentially refine (or even downgrade) long‐term PSE risk classification. However, our dataset cannot address whether early EEG/cEEG meaningfully reclassifies prognosis after suspected/clinical ASyS, nor how monitoring duration should be selected in that population. Finally, although cEEG improved SeLECT‐EEG discrimination and reclassification in this dataset, external and prospective validation is required.

Future work should include duration yield curves and window resampling analyses to test sensitivity to short window placement, center aware modeling, and external validation within the SeLECT consortium. Prospectively, a sequential design is needed in which routine EEG is acquired first and cEEG initiation follows prespecified clinical and electrographic criteria, with harmonized PSE adjudication and explicit evaluation of cost effectiveness and patient centered outcomes.

## Conclusions

In ischemic stroke survivors without ASyS, extending EEG beyond the first hour substantially increases detection of rhythmic/periodic and epileptiform abnormalities – particularly IEDs, LRDA, LPDs, and electrographic seizures – that are frequently missed on a 60‐minute study and are associated with higher long‐term PSE risk. Incorporating cEEG findings improves SeLECT‐EEG discrimination (0.68 vs 0.63) and risk reclassification, indicating that prolonged monitoring adds clinically relevant prognostic information rather than merely increasing detection. Overall, our findings support a selective, phenotype‐ and risk‐guided approach to cEEG monitoring after ischemic stroke, rather than routine application to all patients.

## Author Contributions

KMS and MG contributed to the conception and design of the study; KMS, VD, CT, GN, NG, MG, VP, and CB contributed to the acquisition and analysis of data; KMS and MG contributed to drafting the text or preparing the figures.

## Potential Conflicts of Interest

C.B. reports honoraria for lectures and support for scientific events from Bial, Eisai, and Angelini, outside the submitted work. M.G. reports fees and travel support from Arvelle and Bial, outside the submitted work. The remaining authors have nothing to report.

## Supporting information


**Supplementary Table S1.** Performance of SeLECT_2.0_ Thresholds for Predicting Post‐Stroke Epilepsy (PSE).
**Supplementary Table S2.** Baseline Clinical and EEG Characteristics by PSE.
**Supplementary Table S3.** Comparison of Expected SeLECT‐EEG Point Gain Between SeLECT‐High (≥4) and SeLECT‐Low (<4).
**Supplementary Table S4.** Suggested Prioritization Guide by sEEG Findings – Clinical Detection Yield (Overall), Prognostic PSE Impact if cEEG Newly Detects Regional Slowing or EA, SeLECT‐Based Prioritization, and a Simple Rule of Thumb.
**Supplementary Figure S1.** Overview of Study Cohort, EEG Acquisition, SeLECT‐EEG Predictors, and Four Main Analyses.
**Supplementary Figure S2.** Cumulative Incidence of PSE by Detection Timing on sEEG Versus cEEG—Four‐Group Strata for Epileptiform Activity and Regional Slowing.
**Supplementary Figure S3.** Cumulative Incidence of Late Seizures Stratified by Timing and Presence of EEG Abnormalities.
**Supplementary Figure S4.** Statistical Evaluation of cEEG Prioritization Models.
**Supplementary Figure S5.** Flowchart of Counts in the SeLECT Cohort According to Suggested Prioritization Pathways.

## Data Availability

De‐identified participant data and supporting documentation, including statistical and analytic code, will be made available upon reasonable request following publication. Access will be granted to qualified investigators, contingent upon approval by the lead investigator and the respective local cohort contributors, as well as evidence of appropriate ethical approvals. Requests should be directed to marian.galovic@usz.ch. Data may be used for any purpose deemed scientifically sound and ethically approved.
