# Supplementary material for: Continuous Versus Short EEG After Ischemic Stroke: What cEEG Adds for Detecting Abnormalities and Predicting Post‐Stroke Epilepsy
Source: Ann Neurol. 2026 May 17;100(2):400–15. doi: 10.1002/ana.78251 (PMC13387982; doi:10.1002/ana.78251)
Supplement: Supplementary file 1 — Supplementary Table S1. Performance of SeLECT2.0 Thresholds for Predicting Post‐Stroke Epilepsy (PSE). Supplementary Table S2. Baseline Clinical and EEG Characteristics by PSE. Supplementary Table S3. Comparison of Expected SeLECT‐EEG Point Gain Between SeLECT‐High (≥4) and SeLECT‐Low (<4). Supplementary Table S4. Suggested Prioritization Guide by sEEG Findings – Clinical Detection Yield (Overall), Prognostic PSE Impact if cEEG Newly Detects Regional Slowing or EA, SeLECT‐Based Prioritization, and a Simple Rule of Thumb. Supplementary Figure S1. Overview of Study Cohort, EEG Acquisition, SeLECT‐EEG Predictors, and Four Main Analyses. Supplementary Figure S2. Cumulative Incidence of PSE by Detection Timing on sEEG Versus cEEG—Four‐Group Strata for Epileptiform Activity and Regional Slowing. Supplementary Figure S3. Cumulative Incidence of Late Seizures Stratified by Timing and Presence of EEG Abnormalities. Supplementary Figure S4. Statistical Evaluation of cEEG Prioritization Models. Supplementary Figure S5. Flowchart of Counts in the SeLECT Cohort According to Suggested Prioritization Pathways. [file ANA-100-400-s001.docx]

**Continuous vs. Short EEG after Ischemic Stroke: What cEEG Adds for Detecting Abnormalities and Predicting Poste-stroke Epilepsy**

-- ONLINE SUPPLEMENT --

**Content:**

[Supplemental Methods: Description of Cohorts 2](#_Toc220147937)

[Supplemental Methods: Informed consent procedures 4](#_Toc220147938)

[Supplemental Methods: Other definitions 5](#_Toc220147939)

[Supplemental Table 1: 6](#_Toc220147940)

[Supplemental Table 2 7](#_Toc220147941)

[Supplemental Table 3: 8](#_Toc220147942)

[Supplemental Table 4 9](#_Toc220147943)

[Supplemental Figure 1: 11](#_Toc220147944)

[Supplemental Figure 2: 12](#_Toc220147945)

[Supplemental Figure 3: 13](#_Toc220147946)

[Supplemental Figure 4: 15](#_Toc220147947)

[Supplemental Figure 5: 17](#_Toc220147948)

[Author List and Affiliations of the SeLECT Consortium 18](#_Toc220147949)

# Supplemental Methods: Description of Cohorts

Two-center cEEG cohort (present study). The present analysis pooled consecutive adults with neuroimaging-confirmed acute ischemic stroke from two SeLECT-EEG centers (Hôpital Universitaire de Bruxelles—Hôpital Erasme, Brussels, Belgium; Cleveland Clinic, USA). Source cohort methodologies have been reported previously.^1,2^ After applying this study’s uniform inclusion/exclusion criteria including EEG within 7 days, exclusion of prior seizures/epilepsy, transient ischemic attack, primary hemorrhagic stroke, major epileptogenic comorbidities, and any acute symptomatic seizures per ILAE we analyzed 283 patients (Brussels n=57; Cleveland n=226).

SeLECT consortium registry used for prespecified full-cohort analyses. To contextualize prioritization implications at scale and to derive registry-level estimates, we additionally leveraged the full SeLECT consortium ischemic stroke registry (n=4,552; nine international subcohorts), described in prior SeLECT publications and in the SeLECT-EEG study.^3^ The registry includes adults with neuroimaging-confirmed ischemic stroke and prospectively or retrospectively ascertains early acute symptomatic seizures (≤7 days, ILAE criteria) and remote symptomatic seizures during follow-up, alongside the clinical variables required to compute SeLECT_2.0_. For the present manuscript, registry data were used only to estimate the distribution of SeLECT_2.0_ scores and the proportion of patients meeting our prioritization definitions; consistent with our main analytic cohort, we focused on the acute symptomatic seizure–excluded subset (n=4,319).

**Belgium**

The Belgium cohort involved a retrospective analysis (n=81 stroke survivors) from the prospective continuous EEG (cEEG) and stroke registries at the Hôpital Universitaire de Bruxelles—Hôpital Erasme in Brussels, Belgium. The study included all consecutive adult stroke survivors (≥18 years) who underwent cEEG during the acute ischemic stroke phase between January 1, 2015, and December 31, 2019. cEEG was routinely performed for stroke survivors with non-lacunar supratentorial stroke with an NIHSS score >8, early clinical seizures, or unexplained early neurological deterioration. Stroke survivors with less than one year of follow-up after stroke, prior epilepsy, or epilepsy possibly due to another cause were excluded. The primary outcome of the study was post-stroke epilepsy (PSE), defined as the occurrence of at least one spontaneous seizure more than 7 days after stroke onset. In the present study, 57 of these patients met our harmonized eligibility criteria and were included in the analytic cohort.

Clinical and electrographic data collected included demographics, early clinical seizures, stroke etiology (according to the TOAST classification), admission NIHSS score, stroke territory, and presence of cortical involvement on imaging. Additional data included acute phase treatment and follow-up duration. Outcomes measured were the occurrence of PSE and the modified Rankin Scale scores at the last follow-up, typically obtained during neurovascular, epilepsy, geriatrics, or general neurology follow-up visits.

For EEG analysis, one author, blinded to clinical data and primary outcomes, reviewed the EEG recordings for seizures (either electrographic or electroclinical), highly epileptogenic RPPs (including brief ictal rhythmic discharges, lateralized periodic discharges, bilateral independent periodic discharges and lateralized rhythmic delta activity).^4^ Antiseizure medication was initiated in most stroke survivors with acute symptomatic clinical seizures and tapered during outpatient follow-up in the absence of seizure recurrence.

**USA**

The USA cohort (n=279) was formed utilizing a prospectively maintained stroke and EEG database to identify adults who presented with acute ischemic stroke (AIS) from April 1, 2012, to March 31, 2018. Stroke survivors underwent cEEG monitoring within 7 days of the last known well time, typically indicated for unexplained altered mental status or motor events suggestive of seizure. Each cEEG session began with a preliminary 20-minute EEG screening. The cEEG data and associated clinical records were reviewed by a research associate (LE), who was blinded to the data, to ascertain cases of post-stroke epilepsy (PSE) and matched controls. Stroke survivors with a history of epilepsy prior to AIS were excluded. Those who experienced clinical seizures after hospital discharge, as recorded by their treating physicians, were identified as having PSE. In the present study, 226 of these patients met our harmonized eligibility criteria and were included in the analytic cohort.

The electronic medical records (EMR) were subsequently reviewed to collect acute clinical, neuroimaging, EEG, and anti-seizure medication data. The EEG analysis included looking for epileptiform abnormalities (EAs), such as electrographic seizures based on the Salzburg criteria, ^5,6^ isolated sharp waves (SWs), lateralized periodic discharges (LPDs), lateralized rhythmic delta activity (LRDA), and generalised periodic discharges (GPDs), classified according to the American Clinical Neurophysiology Society nomenclature.^4^

**Summarized Cohort Description Table**

| **Cohort** | **Inception Date** | **Follow-Up Interval** | **EEG Purpose** | **Inclusion Criteria / Outcome Ascertainment Method** |
| --- | --- | --- | --- | --- |
| **Belgium** | 2015-2019 | Median: 35.5 months | Designed to assess EEG post-stroke | Inclusion: Stroke survivors with non-lacunar supratentorial stroke, NIHSS >8, early clinical seizures, or unexplained early neurological deterioration; Exclusion: less than one year of follow-up after stroke, prior epilepsy, or epilepsy possibly due to another cause. Outcome ascertainment via retrospective chart reviews and clinical visits. |
| **USA** | 2012-2018 | Median: 46.7 months | Designed to assess EEG post-stroke | Inclusion: Acute ischemic stroke patients with unexplained altered mental status or a motor event concerning for seizure; Exclusion: Patients with prior epilepsy. Outcome ascertainment via cEEG monitoring and chart reviews. |

**Summarized Cohort Denominators and Attrition Flowchart**

**
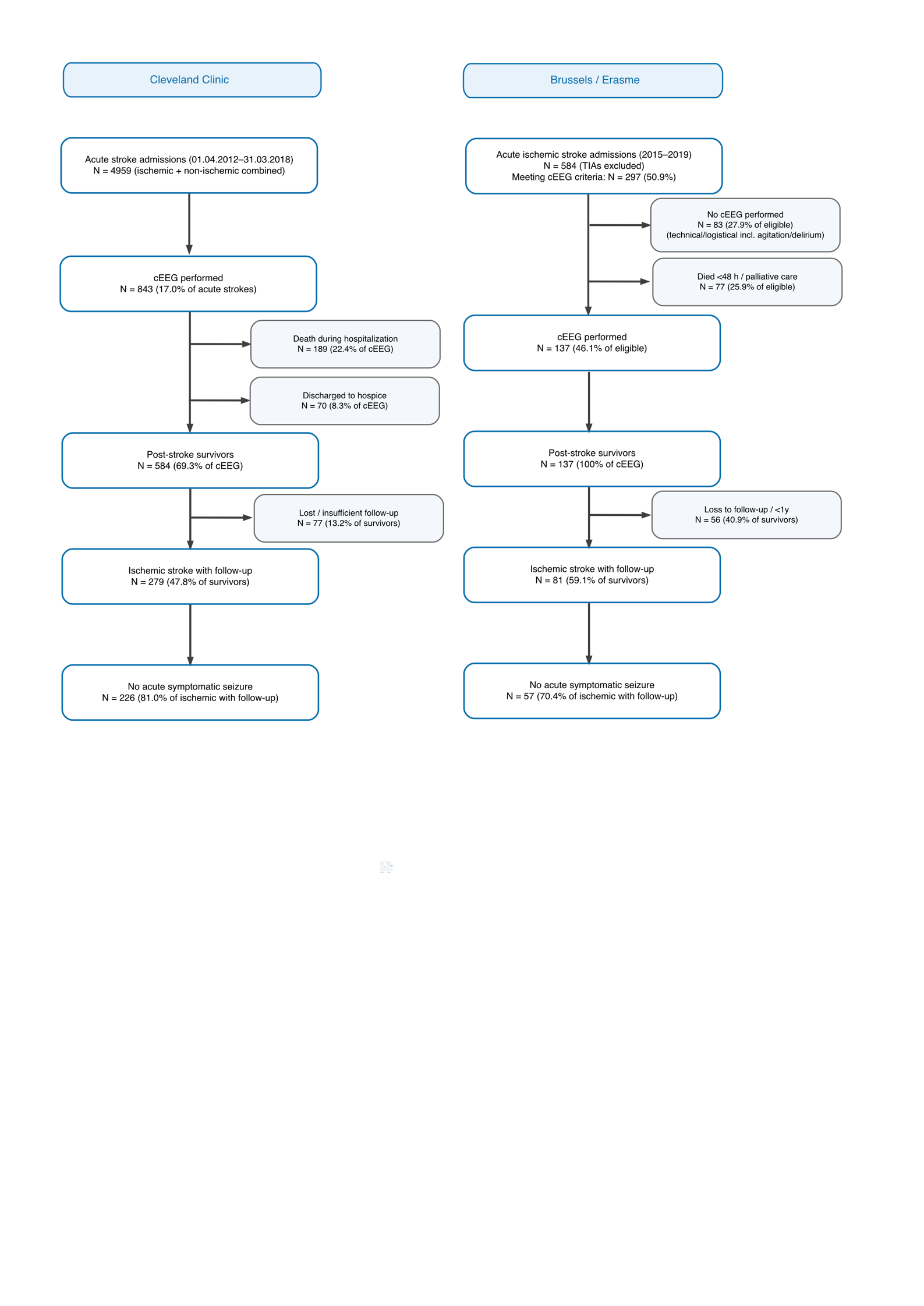
**

# Supplemental Methods: Informed consent procedures

All subjects in the Swiss (2) and Portuguese cohort and those having a face-to-face interview in the Swiss (1) cohort gave written informed consent. All subjects evaluated by telephone in the Swiss (1) cohort gave verbal informed consent. According to Swiss law the regional ethical committees exempted these cohorts from requiring written informed consent. The USA cohort was formed under IRB-approved protocols. The study from the belgian cohort was approved by the Erasme Hospital Ethics Committee, which waived the need for informed consent.

# Supplemental Methods: Other definitions

We used definitions and classifications of the International League Against Epilepsy (ILAE) for seizures types and epilepsy^1-3^ and the World Health Organization for stroke.^4^

Stroke severity was measured with the National Institutes of Health Stroke Scale (NIHSS). Stroke etiology was classified according to the Trial of Org 10172 in Acute Stroke Treatment (TOAST) classification.^5^

# Supplemental Table 1: *Performance of SeLECT2.0 Thresholds for Predicting Post-Stroke Epilepsy (PSE)*

| **SeLECT_2.0_ Threshold** | **Sensitivity** | **Specificity** | **PPV** | **NPV** | **Youden_Index** |
| --- | --- | --- | --- | --- | --- |
| **0** | 100 | 0 | 8.9 | - | 0 |
| **1** | 98.7 | 6.2 | 9.3 | 98 | 4.9 |
| **2** | 96.1 | 21 | 10.6 | 98.2 | 17.1 |
| **3** | 89.6 | 35.5 | 11.9 | 97.2 | 25.1 |
| **4** | 74 | 58.6 | 14.8 | 95.9 | 32.6 |
| **5** | 53.2 | 77.1 | 18.5 | 94.4 | 30.3 |
| **6** | 9.1 | 96.6 | 20.6 | 91.6 | 5.7 |

Supplemental Table 1 summarizes diagnostic performance metrics for various SeLECT2.0 thresholds in predicting late (remote symptomatic) seizures. The optimal balance between sensitivity and specificity was observed at a threshold ≥4, which yielded the highest Youden Index (32.6), supporting its use in the primary rule-based analysis. While lower thresholds maximize sensitivity, they suffer from poor specificity and low positive predictive value (PPV). In contrast, threshold ≥4 maintains high sensitivity (74.0%) with a meaningful gain in specificity (58.6%), yielding the best discriminative performance among all tested cutoffs.

Supplemental Table 2**:** *Baseline clinical and EEG characteristics by PSE*

| **Variable** | **All (n=283)** | **No PSE (n=242)** | **PSE (n=41)** |
| --- | --- | --- | --- |
| Age, years — median (IQR) | 64 (55–74) | 64 (55–75) | 65 (52–74) |
| Sex — n (%) Male | 144 (50.9%) | 122 (50.4%) | 22 (53.7%) |
| Sex — n (%) Female | 139 (49.1%) | 120 (49.6%) | 19 (46.3%) |
| Follow-up, months — median (IQR) | 41 (22–64) | 44 (22–65) | 35 (22–64) |
| Death during follow-up — n (%) | 20 (7.1%) | 14 (5.8%) | 6 (14.6%) |
| NIHSS at admission — ≤3, n (%) | 88 (31.1%) | 79 (32.6%) | 9 (22.0%) |
| NIHSS at admission — 4–10, n (%) | 78 (27.6%) | 72 (29.8%) | 6 (14.6%) |
| NIHSS at admission — ≥11, n (%) | 117 (41.3%) | 91 (37.6%) | 26 (63.4%) |
| MCA territory involvement — n (%) | 214 (75.6%) | 178 (73.6%) | 36 (87.8%) |
| Cortical involvement — n (%) | 196 (69.3%) | 165 (68.2%) | 31 (75.6%) |
| Large-artery atherosclerosis — n (%) | 58 (20.5%) | 45 (18.6%) | 13 (31.7%) |
| All other causes — n (%) | 225 (79.5%) | 197 (81.4%) | 28 (68.3%) |
| EEG pattern on cEEG — ACNS schema |  |  |  |
| Normal EEG | 24 (8.5%) | 24 (9.9%) | 0 (0.0%) |
| Generalized slowing | 116 (41.0%) | 98 (40.5%) | 18 (43.9%) |
| Regional slowing | 166 (58.7%) | 136 (56.2%) | 30 (73.2%) |
| Generalized rhythmic slowing | 101 (35.7%) | 85 (35.1%) | 16 (39.0%) |
| Regional rhythmic slowing (LRDA) | 32 (11.3%) | 24 (9.9%) | 8 (19.5%) |
| GPD | 8 (2.8%) | 7 (2.9%) | 1 (2.4%) |
| LPD | 12 (4.2%) | 6 (2.5%) | 6 (14.6%) |
| IED | 31 (11.0%) | 22 (9.1%) | 9 (22.0%) |
| Electrographic seizure | 12 (4.2%) | 7 (2.9%) | 5 (12.2%) |
| Electrographic status | 1 (0.4%) | 1 (0.4%) | 0 (0.0%) |

Baseline demographic, imaging, and EEG characteristics are summarized for the full cohort (n = 283) and stratified by the occurrence of post-stroke epilepsy (PSE). Continuous variables are reported as median (IQR) and categorical variables as counts (%). EEG findings are shown using ACNS terminology and a mutually exclusive 8-category schema. Compared with patients without PSE (n = 242), those with PSE (n = 41) had more severe strokes at admission (NIHSS ≥ 11: 63.4% vs 37.6%), more frequent MCA territory involvement (87.8% vs 73.6%) and cortical involvement (75.6% vs 68.2%), and a higher proportion with large-artery atherosclerosis (31.7% vs 18.6%). Pathologic EEG features were enriched among PSE, including LPD (14.6% vs 2.5%), IED (22.0% vs 9.1%), and electrographic seizures (12.2% vs 2.9%); whereas normal EEG was absent among PSE (0.0% vs 9.9%).

Abbreviations: PSE, post-stroke epilepsy; cEEG, continuous EEG; ACNS, American Clinical Neurophysiology Society; NIHSS, National Institutes of Health Stroke Scale; MCA, middle cerebral artery; LRDA, lateralized rhythmic delta activity; GPD, generalized periodic discharges; LPD, lateralized periodic discharges; IED, interictal epileptiform discharges.

| **Analysis scope** | **Metric** | **Group N used** | **Mean (High ≥4) – Mean (Low <4)** | **Welch p-value** | **Notes** |
| --- | --- | --- | --- | --- | --- |
| ACNS rows | Expected gain (raw) | 283 | +0.10 | 2.7×10⁻⁴ | Sum of P(new RS)+P(new EA) from cEEG |
| ACNS rows | Gain per possible point | 268 | +0.072 | 9.2×10⁻⁶ | 15 patients excluded (RS+EA already on sEEG ⇒ 0 possible points) |
| ROC (domain-specific) | EA | 250 | AUC 0.54 (0.45–0.64); Youden cut ≈ 3.5 (Sens 0.55 / Spec 0.54) | — | SeLECT weak for predicting *which* new EA occurs |
| ROC (domain-specific) | RS | 168 | AUC 0.57 (0.48–0.66); Youden cut ≈ 2.5 (Sens 0.81 / Spec 0.31) | — | Slightly better than EA, still modest |

# Supplemental Table 3: *Comparison of expected SeLECT-EEG point gain between SeLECT-high (≥4) and SeLECT-low (<4)*

This table summarizes the mean expected SeLECT-EEG point gain from cEEG according to baseline SeLECT_2.0_ risk strata. For the ACNS schema two metrics are shown: the raw expected gain (sum of probabilities of new regional slowing and/or new epileptiform activity on cEEG, given sEEG negative) and the normalized gain per possible point (accounting for ceiling effects; patients already positive for both RS and EA on sEEG had zero possible points and were excluded). Group means, standard deviations, medians, and interquartile ranges are reported, along with Welch t-test comparisons between SeLECT-high (≥4) and SeLECT-low (<4). Results show that SeLECT-high patients consistently exhibited higher incremental yield (≈+0.10 points raw, ≈+0.07 per possible point; both p<0.001), supporting the prespecified ≥4 threshold for cEEG prioritization.

Abbreviations: ACNS = American Clinical Neurophysiology Society; EA = epileptiform activity (including LRDA); RS = regional slowing; GS = generalized slowing; sEEG = short EEG; cEEG = continuous EEG; IQR = interquartile range.

Supplemental Table 4**:** *Suggested prioritization guide by sEEG findings - clinical detection yield (overall), prognostic PSE impact if cEEG newly detects regional slowing or EA, SeLECT-based prioritization, and a simple rule of thumb*

| **sEEG finding** | **Detection of electrographic seizures/status (overall cEEG yield)*** | **Detection of IED/LPD/electrographic seizures/status (overall cEEG yield)*** | **Prognostic impact if cEEG newly detects regional slowing or EA (future PSE) - baseline PSE → incremental SeLECT-EEG gain** | **Rhythmicity / combinations (how to read them)** | **SeLECT effect (how to use it)** | **Rule of thumb (who should get cEEG; purpose clearly stated)** |
| --- | --- | --- | --- | --- | --- | --- |
| **Normal** | ~0–3% | ~3% | Baseline: very low → Gain: Modest (~0.3) | - | SeLECT <4: stays very low. SeLECT ≥4: only modestly higher. | Clinical: — (SeLECT<4), ± if strong concern (SeLECT≥4). Prognostic: gain minimal. |
| **Generalized slowing** | ~2% | ~17% | Baseline: low → Gain: High (~0.5 –0.8) if regional slowing/EA newly found | Often “upgrades” on cEEG to GRDA and/or EA with time | SeLECT ≥4 enriches both detection and prognostic gain | Clinical: + (SeLECT≥4). Prognostic: +++ (SeLECT≥4), ± (SeLECT<4) |
| **GRDA** | Low | ~8% (≈20% if SeLECT≥4) | Baseline: low–mod → Gain: Moderate –High (~0.4–0.6) | Rhythmicity bridges slowing→EA; GRDA+EA raises concern | SeLECT ≥4 meaningfully enriches yield | Clinical: + (SeLECT≥4). Prognostic: +++ (SeLECT≥4), ± (SeLECT<4). |
| **Regional slowing** | ~3% | ~8% (≈12% if SeLECT≥4) | Baseline: modest → Gain: Moderate (~0.3–0.5), mainly via new EA | Watch transition to LRDA - key driver of yield | SeLECT ≥4 moves into “higher risk” zone (for PSE); <4 usually lower | Clinical: + (SeLECT≥4). Prognostic: ++ (SeLECT≥4), ± (SeLECT<4). |
| **LRDA** | ~8% | ~25% | Baseline: intermediate–high → Gain: Low–Moderate (≤~0.3) - EA-like already present; only regional slowing can add | EA-like and often coexists with slowing; monitor for electrographic seizures/status | SeLECT ≥4 further enriches | Clinical: ++ (SeLECT≥4), + (SeLECT<4). Prognostic: ++ (SeLECT≥4), ± (SeLECT<4). |
| **IEDs** | ~22% † | — (already IED/LPD/electrographic seizures/status positive on sEEG) | Baseline: elevated → Gain: Low (0–~0.3) - EA already present; only regional slowing can add | Often with slowing; IED+generalized/regional slowing refines risk level | SeLECT ≥4 increases pre-test probability | Clinical: +++ (SeLECT≥4), ++ (SeLECT<4) to define burden/capture electrographic seizures. Prognostic: + only if regional slowing absent; otherwise ± (little added gain). |
| **LPDs** | ~33% † | — (already IED/LPD/electrographic seizures/status positive on sEEG) | Baseline: very high → Gain: Low (~0–0.2) — EA already present; regional slowing may add little | Mixed EA + slowing common; high vigilance for status | SeLECT ≥4 concentrates highest yield/gain | Clinical: +++ (both strata). Prognostic: + only if regional slowing absent; otherwise ± (adds little). |
| **Electrographic seizure/SE on sEEG** | — (already electrographic seizures/status-positive) | — (already IED/LPD/electrographic seizures/status positive) | Baseline: high by definition → Gain: None (already maxed for EA; regional slowing addition typically minimal) | Often with generalized/regional slowing; status mandates continuous monitoring | SeLECT refines prognosis only; not the monitoring decision | Clinical: +++ (management/trending). Prognostic: n/a (gain none). |

* “Clinical detection” columns reflect immediate therapeutic relevance: electrographic seizures/status drives urgent treatment; IED/LPD/electrographic seizures/status guides anti-seizure therapy/escalation and monitoring strategy.

† Small-n cells for IEDs/LPDs in electrographic seizures/status detection—interpret directionally.

^#^ The timing of detection of epileptiform activity and/or regional slowing within the first seven days after stroke

onset does not seem to affect PSE risk

This table should help to decide who should get cEEG after ischemic stroke. Prioritize the clinical detection columns (electrographic seizures/status and IED/LPD/electrographic seizures/status) - these are immediately therapeutic. The prognostic column is secondary and reports future PSE implications if cEEG newly finds regional slowing /EA (i.e., incremental SeLECT-EEG gain beyond sEEG). If EA is already present on sEEG (e.g., IED/LPD/ electrographic seizures/status), prognostic gain is small to none — cEEG then serves mainly to quantify burden/status, not to add prognostic points. SeLECT ≥4 is a prescreener that shifts many rows “up” (higher yield and/or gain); SeLECT <4 keeps most rows “down” (lower yield/gain). Code used in the Rule column: +++ strongly extend · ++ extend/target · + consider · ± usually no · - no.

Abbreviations: SeLECT, always SeLECT_2.0_ used; SE, status epilepticus.

Supplemental Figure 1: *Overview of study cohort, EEG acquisition, SeLECT-EEG predictors, and four main analyses.*


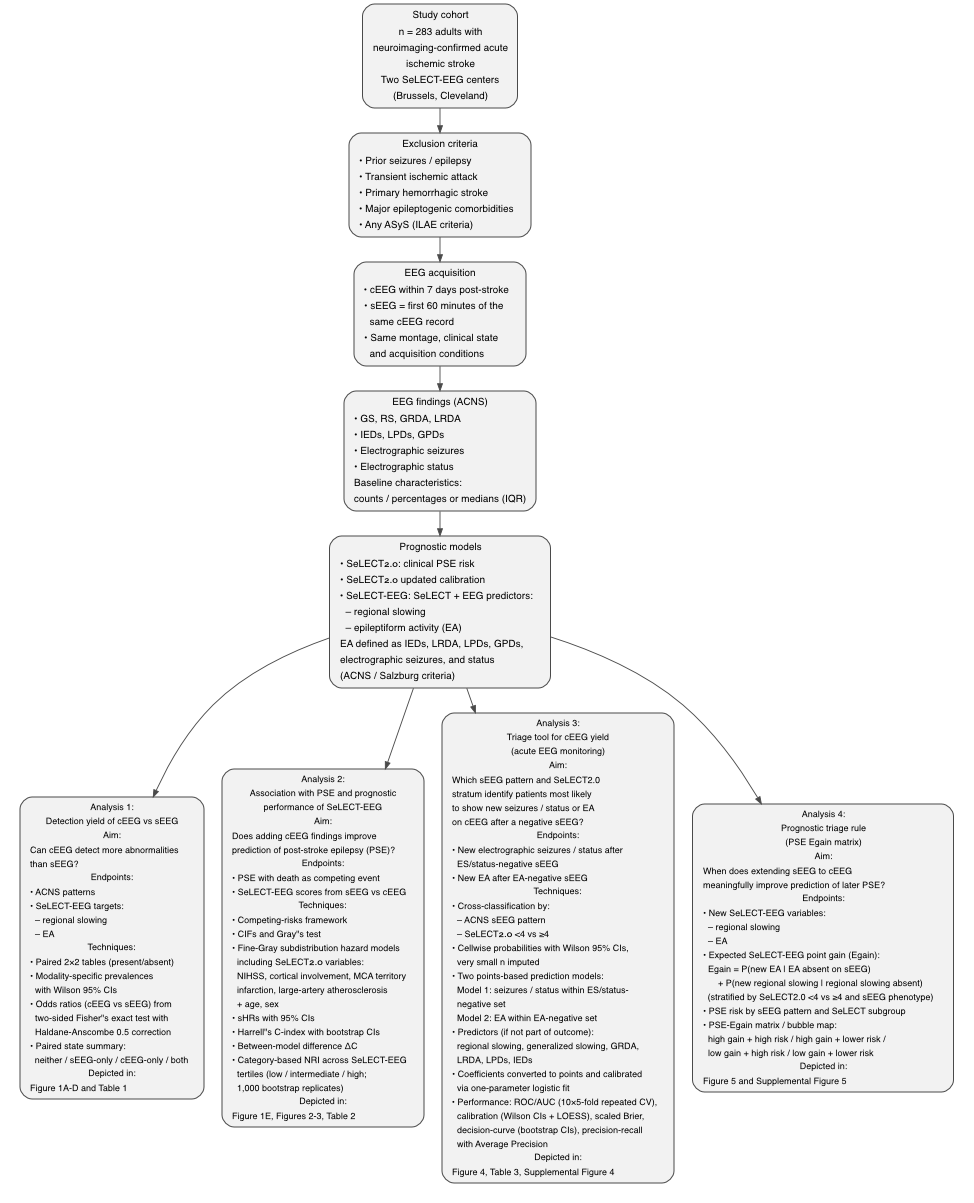


Supplemental Figure 2: *Cumulative incidence of PSE by detection timing on sEEG vs cEEG—four-group strata for epileptiform activity and regional slowing*

*
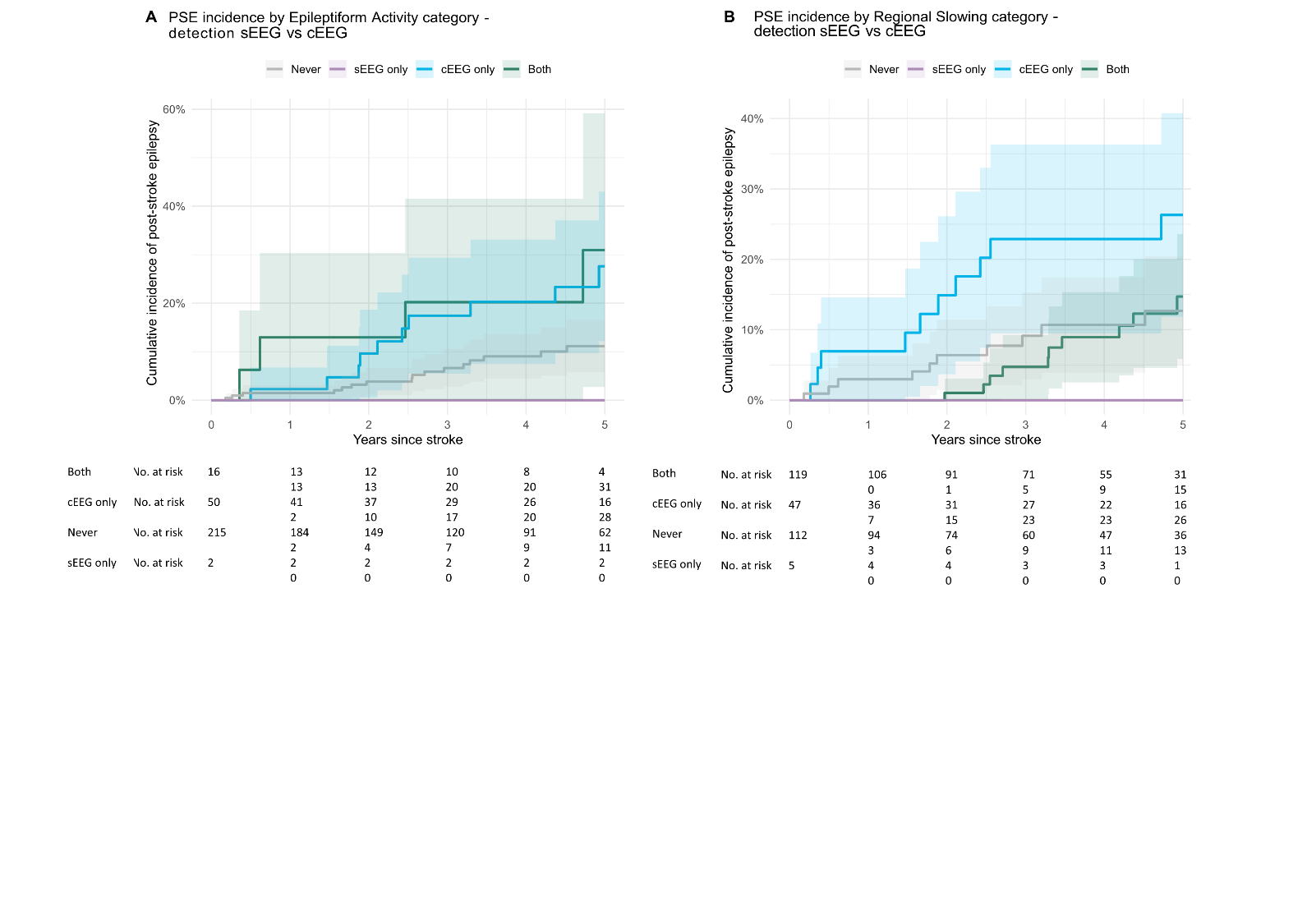
*

Supplemental Figure 2 shows cumulative incidence functions (CIFs) for post-stroke epilepsy (PSE) stratified by detection timing of (A) epileptiform activity (EA) and (B) regional slowing (RS). Groups are defined as “Never” (absent on both recordings), “sEEG only” (present on sEEG but not on cEEG), “cEEG only” (absent on sEEG but present on cEEG), and “Both” (present on both). For EA, PSE risk was highest after 5 years when EA was seen on cEEG—either together with sEEG (“Both,” ≈31%) or only on cEEG (≈28%); no PSE occurred in the “Short only” group, while the “Never” group reached ≈11%. Gray’s test indicated overall differences (p=0.017), driven by a higher risk for “cEEG only” vs “Never” after Holm adjustment (p=0.035). For RS, risk was greatest after 5 years when RS was detected on cEEG only (≈26%), intermediate when persisted on EEG (“Both” ≈15%), and absent in “Short only”; overall Gray’s test p=0.018 (after Holm adjustment 0.18).

Abbreviations: PSE, post-stroke epilepsy; CIF, cumulative incidence function; sEEG, short EEG; cEEG, continuous EEG; EA, epileptiform activity; RS, regional slowing.

Supplemental Figure 3: *Cumulative incidence of late seizures stratified by timing and presence of EEG abnormalities.*


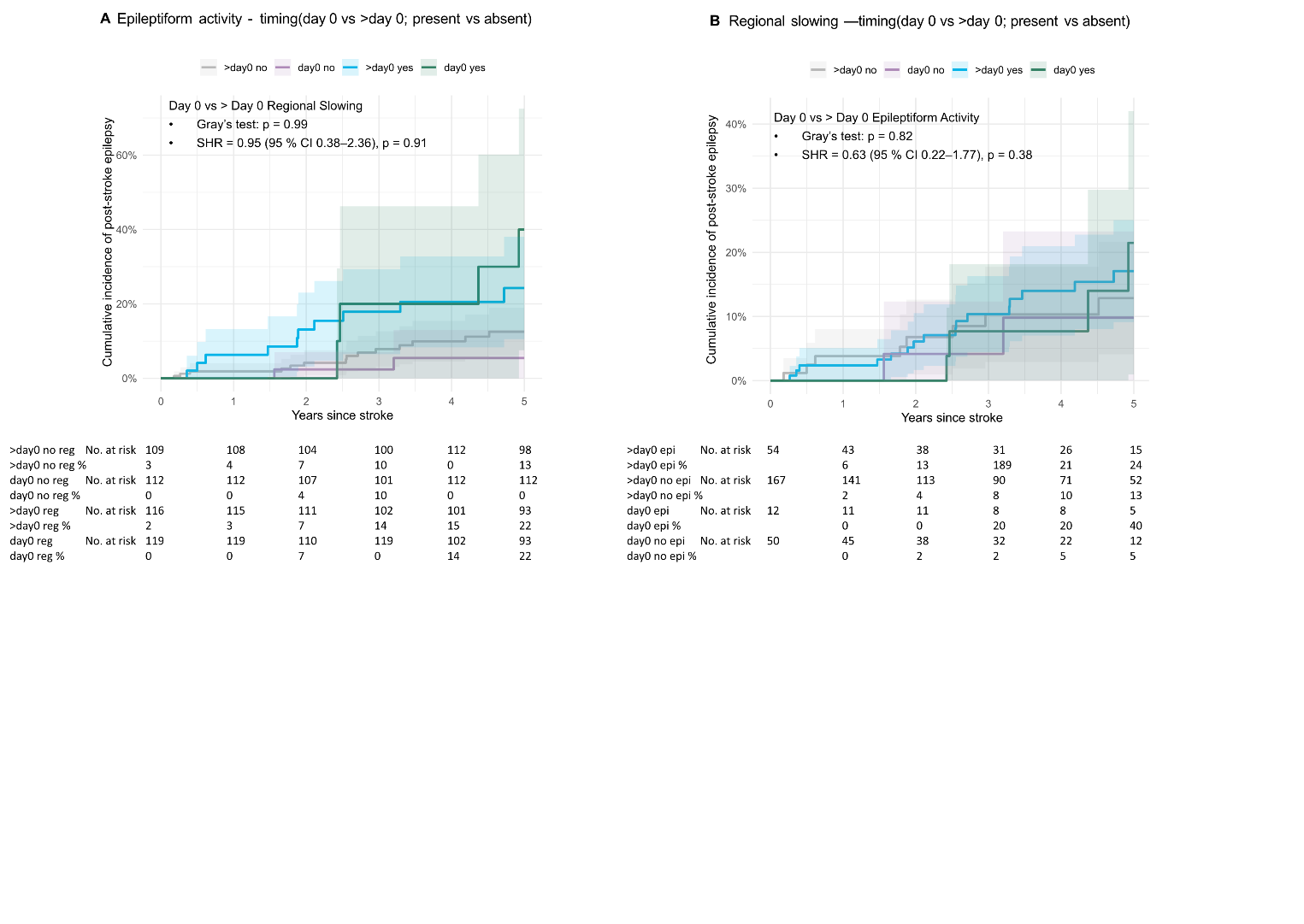


Supplemental Figure 3 shows cumulative incidence functions (competing risk: death) truncated at 5 years and stratified by the presence of the abnormality and the timing of its earliest detection within the first week. Panel A depicts regional slowing (RS); Panel B depicts epileptiform activity (EA). Curves are split into four groups: present on the Day-0 EEG, present only on an EEG performed after Day-0 (within days 1–7), absent on a Day-0 EEG, and absent on an EEG performed after Day-0. Numbers at risk and 5-year cumulative incidences are tabulated below the plots. Across both panels, presence of the abnormality is the primary driver of risk at 5 years. For RS (Panel A), the two present curves sit well above the absent curves by 5 years, but timing alone showed no difference when restricted to those with RS (Day-0 vs >Day-0: Gray’s p = 0.99; Fine–Gray sHR 0.95, 95% CI 0.38–2.36, p = 0.91). A binary comparison of presence vs absence of RS was not significant overall (Gray’s test p≈0.08). For EA (Panel B), 5-year seizure risk is clearly higher when EA is present than when absent; among patients with EA, Day-0 vs >Day-0 timing again did not differ (Gray’s p = 0.82; sHR 0.63, 95% CI 0.22–1.77, p = 0.38). In a binary comparison, presence of EA was associated with higher 5-year seizure incidence than absence (Gray’s p = 0.005). Taken together, within the first week, whether RS or EA is present predicts 5-year PSE risk; when it is first captured (Day-0 vs Days 1–7) does not.

Abbreviations: CIF, cumulative incidence function; PSE, post-stroke epilepsy; EEG, electroencephalography; sEEG, short EEG; cEEG, continuous EEG; Day-0, day of stroke onset; sHR, subdistribution hazard ratio; CI, confidence interval.

discharge; GPD = generalized periodic discharge; IED = interictal epileptiform discharge; LRDA = lateralized rhythmic delta activity.

Supplemental Figure 4: *Statistical evaluation of cEEG prioritization models*


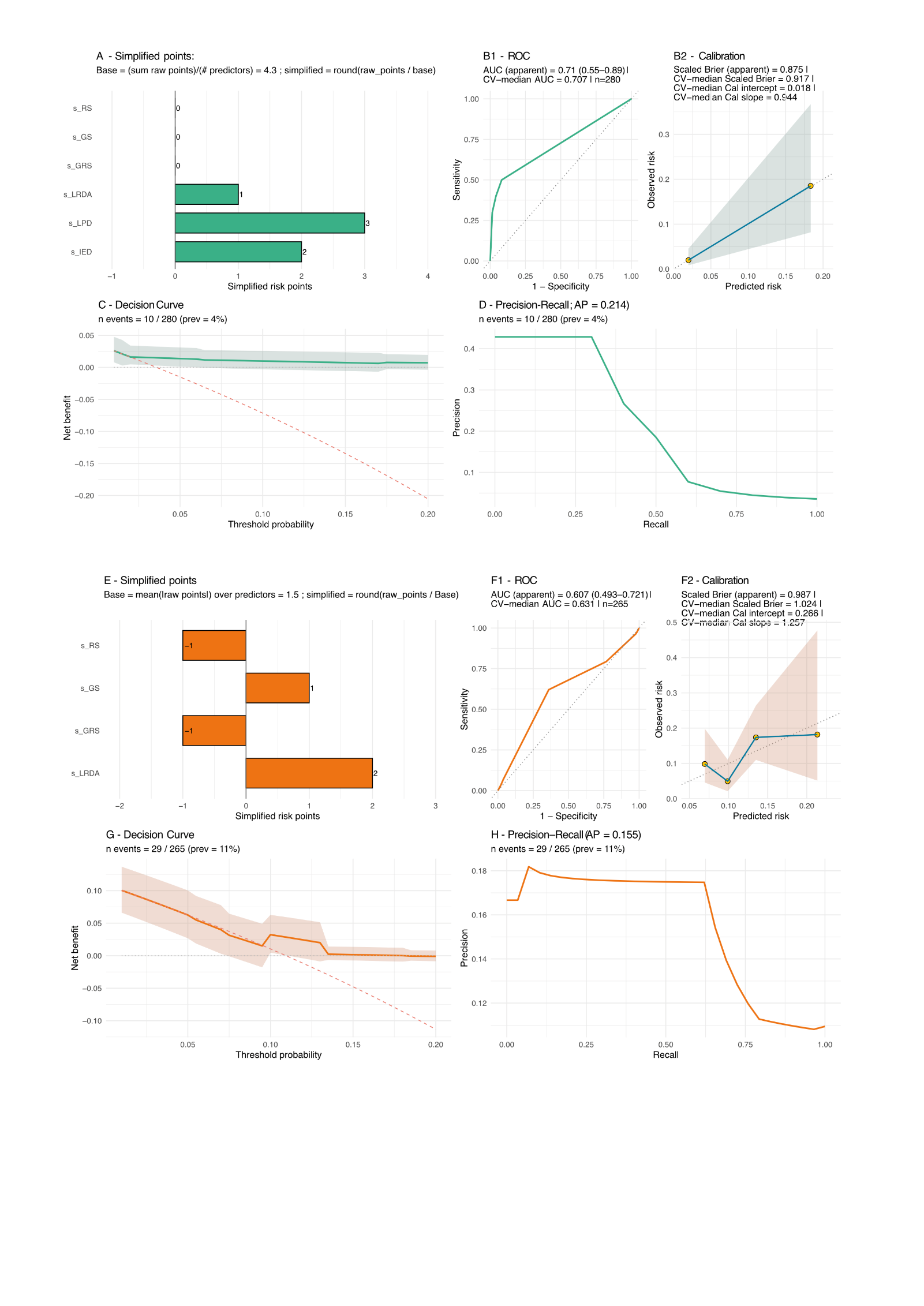


Supplemental Figure 4 presents two statistical evaluations aligned with the heatmap prioritization: a model trained in the sEEG electrographic seizures/status– base to predict new electrographic seizures/status on cEEG using ACNS indicators (RS, GS, GRS, LRDA, LPD, IED; GPD excluded), and a second model trained in the sEEG on a broader set of epileptiform abnormalities – base to predict new epileptiform activity (IED/LPD/electrographic seizures/status) using RS, GS, GRS, LRDA (no LPD/IED/GPD). Logistic coefficients were converted to raw points and then simplified by dividing by the mean absolute raw-points-per-predictor and rounding; performance is shown via ROC (DeLong AUC), calibration (binned observed vs. predicted with Wilson CIs and LOESS smoothing), decision-curve analysis across thresholds 0.01–0.20 with bootstrap 95% CIs, and a precision–recall curve (PRROC) with Average Precision (AP). The electrographic seizures/status model achieves CV-median AUC = 0.71, CV-median scaled Brier = 0.92 (slightly better than a prevalence-only baseline), and CV-median calibration close to ideal (intercept +0.018, slope 0.94); AP ≈ 0.22 versus prevalence 10/280 = 3.6%. On DCA, net benefit is >0 from 0.01–0.20 and exceeds treat-all from ~0.02 onward (treat-none = 0 throughout). The broader epileptiform abnormality model shows CV-median AUC = 0.63, CV-median scaled Brier = 1.024 (≈null baseline), and CV-median calibration with modest over-dispersion (intercept +0.267, slope 1.26); AP ≈ 0.16 versus prevalence 29/265 = 10.9%. Its DCA is >0 from 0.01–0.18 and surpasses treat-all for thresholds ≥0.10 (treat-none = 0).

Abbreviations: cEEG, continuous EEG; sEEG, short EEG; ACNS, American Clinical Neurophysiology Society; ES, electrographic seizure; EAcore, IED/LPD/ES/status composite; IED, interictal epileptiform discharges; LPD, lateralized periodic discharges; LRDA, lateralized rhythmic delta activity; RS, regional slowing; GS, generalized slowing; GRS, generalized rhythmic slowing; ROC, receiver operating characteristic; AUC, area under the curve; PR, precision–recall; AP, average precision; DCA, decision-curve analysis; CI, confidence interval; LOESS, locally weighted regression.

Supplemental Figure 5: *Flowchart of counts in the SeLECT cohort according to suggested prioritization pathways*


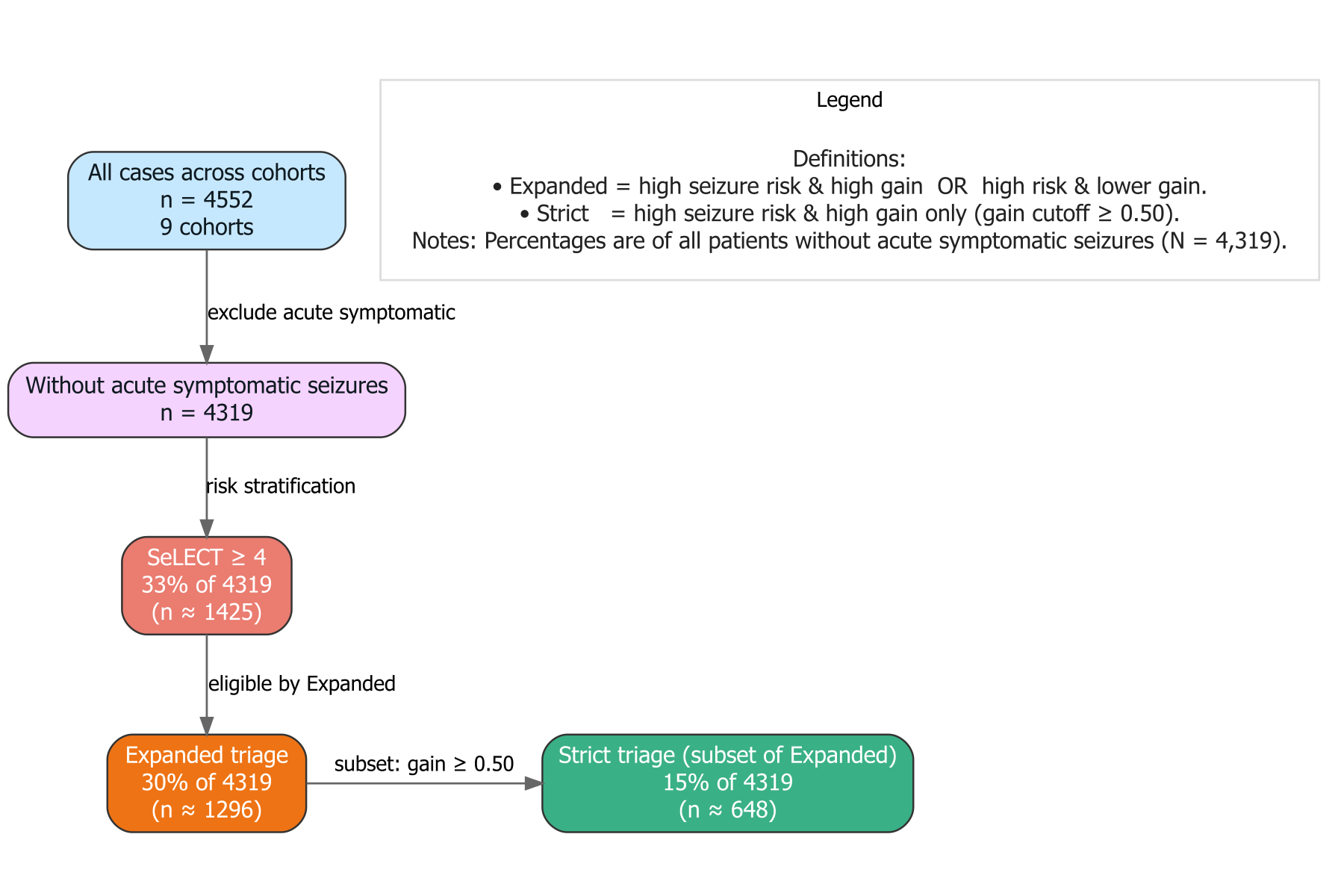


Flowchart summarizing patient selection and prioritization strategies across 9 cohorts (N = 4,552). After excluding acute symptomatic seizures, 4,319 patients remained. Of these, 33% had SeLECT scores ≥4. Within this group, 30% were eligible under the Expanded prioritization definition (high seizure risk with either high or lower gain), and 15% met criteria for Strict prioritization (high seizure risk with high gain only, gain cutoff ≥0.50).

# Author List and Affiliations of the SeLECT Consortium

| **Author** | **Affiliation** |
| --- | --- |
| Andreas Luft, MD | Department of Neurology and Clinical Neuroscience Center, University Hospital Zurich and University of Zurich, Zurich,Switzerland.  Cereneo, Center for Neurology and Rehabilitation & Lake Lucerne Institute, Vitznau, Switzerland. |
| Ana Lúcia Oliveira, MD | Department of Neurosciences and Mental Health (Neurology), Hospital de Santa Maria-ULSSM. Centro de Estudos Egas Moniz, Faculdade de Medicina, Universidade de Lisboa, Lisbon, Portugal |
| Dominik Zieglgänsberger, MD | Department of Neurology, Kantonsspital St. Gallen, St Gallen, Switzerland |
| Giulio Bicciato, MD | Department of Neurology, Clinical Neuroscience Center, University Hospital and University of Zurich, Zurich, Switzerland |
| Laura Abraira, MD PhD | Epilepsy Unit, Department of Neurology, Vall d’Hebron Hospital Universitari, Barcelona; Universitat Autonoma de Barcelona, Bellaterra, Spain |
| Estevo Santamarina, MD | Epilepsy Unit, Department of Neurology, Vall d’Hebron Hospital Universitari, Barcelona; Universitat Autonoma de Barcelona, Bellaterra, Spain |
| José Álvarez-Sabín, PhD | Epilepsy Unit, Department of Neurology, Vall d’Hebron Hospital Universitari, Barcelona; Universitat Autonoma de Barcelona, Bellaterra, Spain |
| Carolina Ferreira-Atuesta, MD MSc | Department of Clinical & Experimental Epilepsy, UCL Queen Square Institute of Neurology, London WC1N 3BG & Chalfont Centre for Epilepsy, Chalfont St Peter SL9 0RJ, United Kingdom; Department of Neurology, Icahn School of Medicine at Mount Sinai, New York, United States |
| Mira Katan, MD MSc | Department of Neurology, Clinical Neuroscience Center, University Hospital and University of Zurich, Zurich, Switzerland; Department of Neurology, University Hospital and University of Basel, Basel, Switzerland |
| Nico Döhler, MD | Department of Neurology, Kantonsspital St. Gallen, St Gallen, Switzerland; Specialist Clinic for Neurorehabilitation, Kliniken Beelitz, Beelitz-Heilstätten, Germany |
| Adam Strzelczyk, MD | Epilepsy Center Frankfurt Rhine-Main, Department of Neurology, Goethe-University Frankfurt, Frankfurt am Main, Germany. |
| Barbara Erdélyi-Canavese, MD | Department of Neurology, Kantonsspital St. Gallen, St Gallen, Switzerland |
| Ansgar Felbecker, MD | Department of Neurology, Kantonsspital St. Gallen, St Gallen, Switzerland; Department of Neurology, University Hospital Inselspital Bern, Bern, Switzerland |
| Philip Siebel, MD | Department of Neurology, Kantonsspital St. Gallen, St Gallen, Switzerland |
| Michael Winklehner, MD | Johannes Kepler University Linz, Kepler University Hospital, Department of Neurology, Altenberger Straße 69, 4040 Linz and Wagner-Jauregg Weg 15, 4020 Linz, Austria |
| Tim J von Oertzen, MD FRCP | Johannes Kepler University Linz, Kepler University Hospital, Department of Neurology, Altenberger Straße 69, 4040 Linz and Wagner-Jauregg Weg 15, 4020 Linz, Austria |
| Judith N. Wagner, MD | Johannes Kepler University Linz, Kepler University Hospital, Department of Neurology, Altenberger Straße 69, 4040 Linz and Wagner-Jauregg Weg 15, 4020 Linz, Austria; Department of Neurology, Evangelisches Klinikum Gelsenkirchen, Academic Hospital University Essen-Duisburg, Gelsenkirchen, Germany |
| Giovanni Merlino, MD PhD | Department of Medicine, University of Udine and Clinical Neurology, Udine University Hospital, Udine, Italy. |
| Mariarosaria Valente, MD | Department of Medicine, University of Udine and Clinical Neurology, Udine University Hospital, Udine, Italy. |
| Gian Luigi Gigli, MD | Department of Medicine, University of Udine and Clinical Neurology, Udine University Hospital, Udine, Italy |
| Annacarmen Nilo, MD | Department of Medicine, University of Udine and Clinical Neurology, Udine University Hospital, Udine, Italy |
| Francesco Janes, MD PhD | Department of Medicine, University of Udine and Clinical Neurology, Udine University Hospital, Udine, Italy |
| Alessandra Burini, MD | Department of Medicine, University of Udine and Clinical Neurology, Udine University Hospital, Udine, Italy |
| Domenico Maisano, MD | Department of Medicine, University of Udine and Clinical Neurology, Udine University Hospital, Udine, Italy |
| María Paula Zafra-Sierra, MD | Department of Neurology, Fundación Santa Fe de Bogotá, Universidad de Los Andes, Universidad del Bosque, Bogotá, Colombia |
| Luis Carlos Mayor-Romero, MD | Department of Neurology, Fundación Santa Fe de Bogotá, Universidad de Los Andes, Universidad del Bosque, Bogotá, Colombia |
| Julian Conrad, MD | Department of Neurology, University of Muenster, Muenster, Germany; Division for neurodegenerative diseases, Department of Neurology, Universitaetsmedizin Mannheim, University of Heidelberg |
| Stefan Evers, MD PhD | Department of Neurology, University of Muenster, Muenster, Germany; Department of Neurology, Krankenhaus Lindenbrunn, Coppenbrügge, Germany |
| Piergiorgio Lochner, MD | Department of Neurology, Saarland University Medical Center, Homburg, Germany |
| Frauke Roell, MD | Department of Neurology, Saarland University Medical Center, Homburg, Germany |
| Francesco Brigo, MD | Department of Neurology, Hospital of Merano (SABES-ASDAA), Merano-Meran, Italy |
| Mark R Keezer, MDCM PhD | Department of Clinical & Experimental Epilepsy, UCL Queen Square Institute of Neurology, London WC1N 3BG & Chalfont Centre for Epilepsy, Chalfont St Peter SL9 0RJ, United Kingdom; Centre Hospitalier de l’Université de Montréal, Montreal, QC, Canada |
| John S Duncan, FRCP FMedSci | Department of Clinical & Experimental Epilepsy, UCL Queen Square Institute of Neurology, London WC1N 3BG & Chalfont Centre for Epilepsy, Chalfont St Peter SL9 0RJ, United Kingdom |
| Josemir W Sander, FRCP FMedSci | Department of Clinical & Experimental Epilepsy, UCL Queen Square Institute of Neurology, London WC1N 3BG & Chalfont Centre for Epilepsy, Chalfont St Peter SL9 0RJ, United Kingdom; Stichting Epilepsie Instellingen Nederland –(SEIN), Heemstede 2103 SW, The Netherlands |
| Barbara Tettenborn, MD | Department of Neurology, Kantonsspital St. Gallen, St Gallen, Switzerland |
| Matthias J Koepp, MD PhD | Department of Clinical & Experimental Epilepsy, UCL Queen Square Institute of Neurology, London WC1N 3BG & Chalfont Centre for Epilepsy, Chalfont St Peter SL9 0RJ, United Kingdom |

# References

1. Tatillo C, Legros B, Depondt C, et al. Prognostic value of early electrographic biomarkers of epileptogenesis in high-risk ischaemic stroke patients. *Eur J Neurol*. 2024;31(1):e16074. doi:10.1111/ene.16074

2. Punia V, Ellison L, Bena J, et al. Acute epileptiform abnormalities are the primary predictors of post-stroke epilepsy: a matched, case-control study. *Ann Clin Transl Neurol*. 2022;9(4):558-563. doi:10.1002/acn3.51534

3. Schubert KM, Dasari V, Oliveira AL, et al. The Role of Electroencephalography in Predicting Post‐Stroke Seizures and an Updated Prognostic Model ( SeLECT ‐ EEG ). *Annals of Neurology*. 2025;98(4):814-825. doi:10.1002/ana.27301

4. Hirsch LJ, Fong MWK, Leitinger M, et al. American Clinical Neurophysiology Society’s Standardized Critical Care EEG Terminology: 2021 Version. *J Clin Neurophysiol*. 2021;38(1):1-29. doi:10.1097/WNP.0000000000000806

5. Trinka E, Cock H, Hesdorffer D, et al. A definition and classification of status epilepticus--Report of the ILAE Task Force on Classification of Status Epilepticus. *Epilepsia*. 2015;56(10):1515-1523. doi:10.1111/epi.13121

6. Leitinger M, Trinka E, Gardella E, et al. Diagnostic accuracy of the Salzburg EEG criteria for non-convulsive status epilepticus: a retrospective study. *Lancet Neurol*. 2016;15(10):1054-1062. doi:10.1016/S1474-4422(16)30137-5
